# Controlling the activity of quorum sensing autoinducers with light†
†Electronic supplementary information (ESI) available: Detailed experimental information on chemical synthesis, photoswitching experiments, bacterial growth conditions, bioluminescence assay, RNA isolation and cDNA synthesis, gene expression and pyocyanine assay details. See DOI: 10.1039/c5sc00215j


**DOI:** 10.1039/c5sc00215j

**Published:** 2015-04-27

**Authors:** J. P. Van der Berg, W. A. Velema, W. Szymanski, A. J. M. Driessen, B. L. Feringa

**Affiliations:** a Molecular Microbiology , Groningen Biomolecular Sciences and Biotechnology Institute , University of Groningen , Nijenborgh 7, 9747 AG , Groningen , The Netherlands . Email: a.j.m.driessen@rug.nl; b Center for Systems Chemistry , Stratingh Institute for Chemistry , University of Groningen , Nijenborgh 4, 9747 AG , Groningen , The Netherlands . Email: b.l.feringa@rug.nl; c Department of Radiology , University of Groningen , University Medical Centre Groningen , Groningen , The Netherlands

## Abstract

Bacteria use a communication system, called quorum sensing (QS), to organize into communities and synchronize gene expression to promote virulence and secure survival. Here we report on a proof-of-principle for externally interfering with this bacterial communication system, using light. By employing photoswitchable small molecules, we were able to photocontrol the QS-related bioluminescence in an *Escherichia coli* reporter strain, and the expression of target QS genes and pyocyanin production in *Pseudomonas aeruginosa*.

## Abstract 2

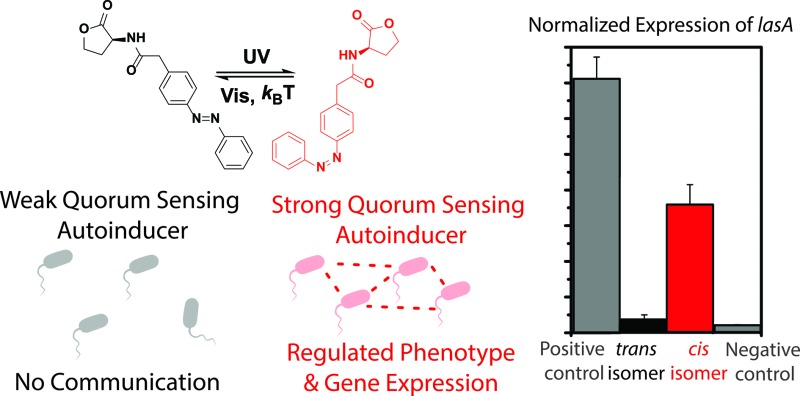
Bacteria use Quorum Sensing (QS) to organize into communities and synchronize gene expression. Here we report on a method to externally interfere with QS system using light.

## Introduction

Like higher organisms, bacteria are able to organize themselves in multicellular groups, which offers the resulting bacterial communities certain advantages, such as a greater resistance against host defence mechanisms and an improved antibiotic tolerance, as compared to the individual cells.^1^ In order to organize, bacteria rely on quorum sensing (QS) as a system of communication, which is dependent on the population density.^2^ Using QS, bacteria are able to synchronize their gene expression and regulate their pathogenicity, biofilm formation and fitness,^1^,^3^ amongst others. Establishing external control over QS is challenging, but might be highly useful to regulate bacterial organization. Not only is it a viable possibility to regulate gene expression^4^ in a large population of bacteria, it can also be considered an important alternative as a therapeutic strategy.^5^,^6^

Light, which can be delivered with high spatiotemporal resolution, has successfully been employed to gain control over biological function, as was shown in the fields of optogenetics^7^ and photopharmacology.^8^ The molecular approaches rely on the use of photocaged^9^ and photoswitchable small molecules.^10^–^13^ In particular, photopharmacology relies on the introduction of photoresponsive units in the structure of bio-active compounds.^14^ By irradiating with different wavelengths of light, the structure and properties of the bio-active compound can be reversibly switched between two or more stages, altering the physiological properties of the compound.^8^ In the bacterial QS system, small molecules, called auto-inducers, act as signals that can be detected by receptors.^3^,^15^ We envisioned that the introduction of a molecular photoswitch in the structure of such a signal molecule might allow for external regulation over QS-related gene expression and bacterial phenotypes, which offers prospects for manipulating bacterial group biology.

Here, we describe the design, synthesis and biological activity of three photoswitchable QS signalling molecules, incorporating an azobenzene photochromic unit. Two of the signalling molecules are shown to have an opposite effect upon UV-light irradiation in bioluminescence assays, *i.e.* one molecule gains QS-inducing activity and the other loses its activity, upon *trans*–*cis* photoisomerization of the azobenzene unit. Furthermore, the reported compounds allow the photochemical control over the expression of virulence genes in *Pseudomonas aeruginosa.* Finally, we show the optical control over bacterial phenotype by regulating pyocyanin production. These experiments constitute a new approach to interfere, in a non-invasive manner, with bacterial communication and have potential to control bacterial group biology. In addition, this method might be useful to tackle bacterial pathogenesis and study QS. It represents a promising tool for biotechnology, taking advantage of the potential to externally regulate the expression of a wide range of target genes.

## Results and discussion

### Design of photoswitchable autoinducers

**Fig. 1:**
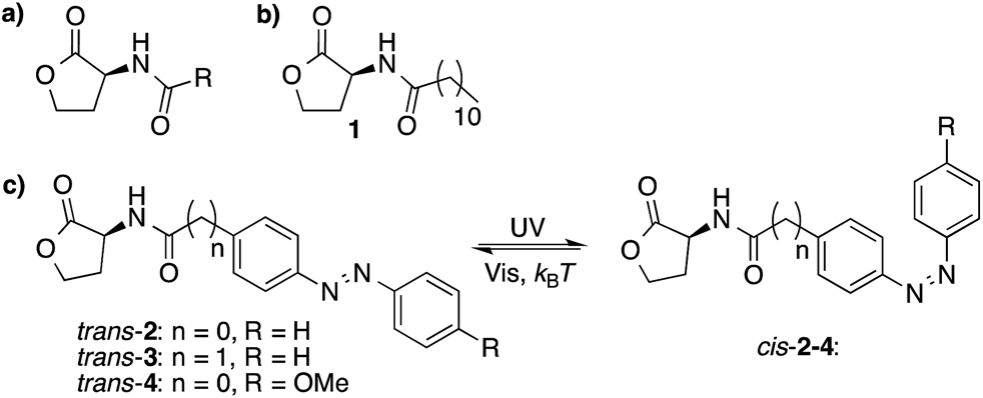
Molecular structures of AHLs. (a) The structure of naturally-occurring AHLs, where R is usually an alkyl chain varying in length between 4 and 18 carbon atoms. (b) Structure of a naturally occurring auto-inducer (**1**) (c) Three photoswitchable analogs (**2**, **3** and **4**) that can undergo *trans*–*cis* photoisomerization upon UV-light irradiation and *cis*–*trans* isomerization upon visible-light irradiation and thermal relaxation.

*N*-Acyl homoserine lactones (AHLs) are an important class of QS auto-inducers that play a major role in the QS system of Gram-negative bacteria.^3^ These molecules consist of a *N*-acyl homoserine lactone moiety and an aliphatic chain of varying length (Fig. 1a). The molecular design used here is based on compound **1** (Fig. 1b), which has been reported to have QS-inducer activity in a broad range of bacteria,^16^ including *Burkholderia cenocepacia*,^17^*Chromobacterium violaceum*,^18^*Pseudomonas aeruginosa*,^19^*Sinorhizobium meliloti*^19^ and *Vibrio fischeri*.^20^,^21^ Co-crystal structures of the 3-oxo analog of compound **1**, bound to its receptor LasR, reveal that all the AHLs heteroatoms, except for the oxygen in the lactone ring, form hydrogen bonds with the receptor protein, and that the alkyl chain is located in a hydrophobic cavity.^22^

Azobenzenes are a class of photochromic compounds that can be switched between a *trans* and *cis* form with light, which is accompanied by a large change in polarity and geometry. Therefore, we envisioned that the introduction of a photoresponsive azobenzene moiety into the hydrophobic part of the QS autoinducer,^23^ as has been suggested by Blackwell and coworkers,^24^ might block the interaction between the ligand and its receptor in one of the photoisomeric states, whereas the activity would be retained in the other isomeric form. Further support for this hypothesis comes from recent studies showing that the activity of autoinducers was conserved after introduction of an aromatic ring in the aliphatic part of the molecule.^16^

Three molecules (**2–4**, Fig. 1c) were designed with an azobenzene moiety conjugated to a *N*-acyl homoserine lactone. Compound **3** differs from compound **2** by the addition of an extra methylene group. The presence of sterically bulky and rigid groups in the acyl side chain have been shown to have a large effect on QS activity,^24^ therefore the methylene group was introduced to offer more flexibility to the hydrophobic part of the molecule (Fig. 1c). Compound **4** bears an additional *p*-methoxy group, which has been reported to cause a large difference in *trans–cis* ratio of the azobenzene moiety, before and after 365 nm light irradiation (*vide infra*).^8^

### Photoswitchable behaviour of autoinducers

The azobenzene-containing molecules consist of a mixture of *trans* and *cis* isomers. The ratio between the two isomers can be changed by irradiation with light (Table 1).^23^ Upon UV-light irradiation, the *trans* molecules undergo a photochemical isomerization, changing the ratio to mostly *cis*-isomer.

**Table 1:** *Trans*–*cis* ratios of compound **2**, **3** and **4** before and after *λ* = 365 nm light irradiation in DMSO_d6_ at room temperature and half-lives of the *cis*-isomer at 30 °C and 37 °C in water

Compound	**2**	**3**	**4**
No irradiation (DMSO_d6_, *trans*–*cis*)	97 : 3	97 : 3	97 : 3
365 nm light irradiation (DMSO_d6_, *trans*–*cis*)	38 : 62	10 : 90	4 : 96
Half-life in H_2_O at 30 °C (hours)	>10	6.8	8.9
Half-life in H_2_O at 37 °C (hours)	>10	2.5	5.8

Subsequent exposure to visible light changes the ratio back to mostly *trans*-isomer. This last step can also be achieved by thermal relaxation, because the *cis*-isomer is the thermodynamically less stable form. Table 1 shows the *trans*–*cis* ratios of compound **2**, **3** and **4** before and after *λ* = 365 nm light irradiation in DMSO, which were determined using ^1^H NMR spectroscopy (Fig. S1–S3†). The UV-Vis absorption spectra of compound **2**, **3** and **4** show an absorption maximum around 340 nm, which is characteristic of *trans* azobenzene.^23^,^25^ Upon irradiation with *λ* = 365 nm light, this absorption maximum decreases, which is typical for *trans*–*cis* azobenzene isomerization (Fig. S4–S6†).^23^,^25^ By monitoring the recovery of the absorbance at this maximum at 37 °C in water overtime, the half-life of the *cis* isomer of compounds **2**, **3** and **4** were determined (Table 1 and Fig. S7–S12†), showing that the *cis*-isomers are rather stable, with half-lives in the multi-hour range. Because activity assays (*vide infra*) contain an incubation step of less than two hours, only minor thermal *cis*–*trans* isomerization is expected to occur while assaying the activity of the 365 nm light-irradiated compounds.

### Bioluminescence activity assay

The major QS signalling network in *P. aeruginosa* is the Las system. This system is regulated by the level of AHLs that are produced by the signal molecule synthase LasI. Activation of the signalling network requires a sufficient threshold concentration of AHLs, which is coincident with a high cell density.^1^,^3^ Before the threshold concentration of the AHLs is reached, the transcriptional activator LasR will be rapidly degraded due to incorrect folding. Upon AHL binding, LasR can fold properly and form a stable dimer,^22^ which can bind responsive promoter regions on the bacterial genome and thereby activate transcription of QS-controlled genes. Virulent genes regulated by the LasQS include *lasI*, *lasA*, and *lasB*, but also other QS systems, like Rhl QS, are controlled by the Las system.^26^ To measure the effect of the photoswitchable AHL molecules on the Las quorum sensing system, *E. coli* JM109 pSB1075 was used. Addition of LasQS ligands to this biosensor strain results in a rapid emission of bioluminescence due to the presence of a *luxCDABE-lasR* promoter fusion.^27^

**Fig. 2:**
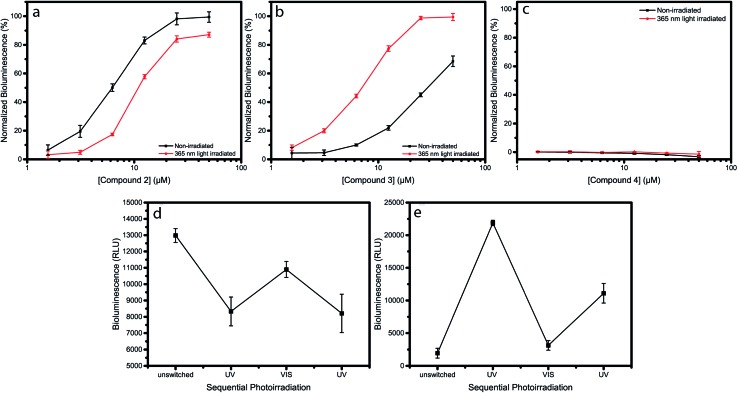
Dose–response curves of compounds **2**, **3** and **4**, before and after irradiation, obtained by measuring the LasQS controlled bioluminescence at different concentrations, using the *E. coli* JM109 pSB1075 sensor strain. Dose–response curve of non-irradiated (black) and *λ* = 365 nm light-irradiated (red) compounds **2** (a), **3** (b) and **4** (c). (d) and (e) Effect of photoswitching on the biological activity of compounds **2** (d) and **3** (e). Both compounds were used at a concentration of 6.25 μM for the sequential rounds of photoirradiation. Data points and error bars represent the mean and pooled standard deviation of two biological replicates each performed in triplicate.

Indeed, addition of compound **1** to the *E. coli* sensor strain resulted in an increase in luminescence after approximately 1–2 hours of incubation (Fig. S13†). When compound **2** or **3** were added to the reporter strain, *E. coli* JM109 pSB1075, a dose-dependent increase in bioluminescence was observed (Fig. 2a and b), while addition of compound **4** did not result in any increase in bioluminescence (Fig. 2c). To examine the effect of photoswitching on the activity of the compounds, the same experiment was repeated, but now the compounds were exposed to 365 nm light for 5 min, prior to incubation. A drop in efficacy, as well as a decrease in potency, were observed for compound **2**. This indicates that *cis*-**2** activates the LasQS pathway less effectively, as compared to *trans*-**2** (Fig. 2a). Surprisingly, the opposite, and an even more strongly-pronounced effect was observed for compound **3**: after UV irradiation at 365 nm, an almost 5-fold increase in activity was observed, implying that, in its *cis*-isomeric form, compound **3** more effectively activates the Las system as compared to *trans*-**3** (Fig. 2b). After *λ* = 365 nm light irradiation, compound **4** still did not exhibit any significant effect (Fig. 2c). SAR studies have shown that rigid bulky groups in the acyl side chain can decrease the activity of autoinducers.^24^ The observed opposite change in activity after photoisomerization of compound **2** and **3**, can possibly be explained by the conformation the autoinducers adopt.

*Trans*-**2** and *cis*-**3** both exhibit linear shapes, which might allow for a more optimal interaction with the hydrophobic binding pocket of the receptor protein, whereas *cis*-**2** and *trans*-**3** have a more bended structure, hindering interactions with the binding pocket and resulting in a lower activity.

The lack of activity of compound **4** might be attributed to the presence of the more hydrophilic methoxy group that disrupts the hydrophobic interactions between the acyl side chain and the hydrophobic cavity in the binding pocket of the receptor protein.

### Photochemical stability of autoinducers

Next, we addressed the issue of the photochemical stability of the switchable autoinducers, by investigating the reversibility of QS induction by applying sequential rounds of photoirradiation with *λ* = 365 nm light, followed by white light, on samples of **2** and **3**. After each irradiation step, the biological activity was evaluated. This excludes the possibility that UV-irradiation leads to irreversible photochemical degradation to products that would show different QS-inducing behavior and might influence the experiments presented in Fig. 2. Irradiation of compound **2** with *λ* = 365 nm light results in switching to the *cis*-isomer, which has a lower QS inducing activity and its addition led to lower bioluminescence. Sequential exposure to white light switched compound **2** to the *trans*-isomer, which has higher QS-inducing activity, and resulted in an increased bioluminescence. Subsequent exposure to *λ* = 365 nm light again resulted in a decreased luminescence (Fig. 2d). Similar rounds of irradiation of compound **3** resulted in opposite behavior (Fig. 2e). These results show how compounds **2** and **3** are able to switching the QS system “ON and OFF” in an opposite fashion, although the maximum reached bioluminescence decreases after each round of photoswitching. We attribute this to the instability of the lactone ring, since the azobenzene switch itself shows little or no fatigue upon alternating irradiation with UV and white light (Fig. S14–S16†). This experiment gives insight into the stability of the photoswitchable autoinducers upon multiple cycles of light irradiation. Additionally, it emphasizes the reproducibility of the observed differences in activity between both isomers.

In the described experiments, the irradiation was performed prior to incubation, due to the toxicity of UV light to bacteria. UV light is known to cause DNA lesions in bacteria and therefore direct exposure should be avoided.^28^ However, direct irradiation of the bacteria, when not toxic, would allow for rapidly increasing and decreasing the active inducer concentration, which might lead to new photocontrolled tools for studying bacterial group biology. Recent developments towards photochromic systems that can be addressed with non-toxic visible light^29^–^33^ might eventually be adopted for our approach and lead to systems that can be illuminated during bacterial growth. For example, Woolley and co-workers reported^29^ red-light switchable azobenzenes that were obtained by introduction of methoxy groups in all *ortho* positions of the azobenzene. Such a structure might be adopted to obtain red-light switchable autoinducers and would allow the irradiation of bacterial cultures.

### Photocontrol of gene expression

**Fig. 3:**
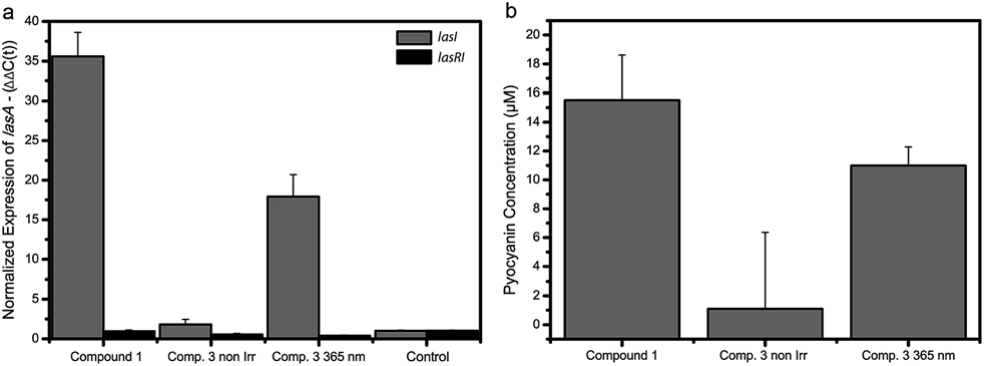
Gene expression and pyocyanin production in *P. aeruginosa*. (a) Effect of compounds **1** (10 μM) and **3** (100 μM) on the gene expression of *las*-controlled genes in *P. aeruginosa*. Gene expression of *lasA* in the presence of non-irradiated compound **3**, *λ* = 365 nm irradiated **3** and compound **1** in the Δ*lasI* (gray) and Δ*lasRI* (black) strains. All samples are compared to the control sample. Data is plotted as mean and standard deviation of two biological replicates each performed in duplo. (b) The effect of compounds **1** (50 μM) and **3** (2 × 50 μM) on pyocyanin production in cultures of *P. aeruginosa* Δ*lasI* before and after *λ* = 365 nm light-irradiation. Data is plotted as mean and standard deviation of three experiments consisting of two biological replicates.

It has been shown that the LasQS system in *P. aeruginosa* is responsible for the regulation of several downstream mechanisms, including virulence.^34^*Las*A is one of the many genes controlled by LasQS and encodes the protease LasA that has bacteriolytic activity and enhances the elastinolytic activity of other proteases.^35^ We measured the effect of the photoswitchable signal molecules on the QS-controlled gene *lasA* in *P. aeruginosa*, in order to examine if these compounds can be used to manipulate the expression of QS-regulated genes *in vivo*. Compound **3** was chosen for these studies, because it showed the largest difference in activity between the non-irradiated and *λ* = 365 nm light-irradiated forms in the bioluminescence assay (Fig. 2b). For this purpose, the “signal negative” *P. aeruginosa* PA14 Δ*lasI* strain was used, which is defective in the production of LasQS AHL molecules. Since QS synchronizes gene expression at a high cell density, *P. aeruginosa* were grown to a late exponential growth phase before adding synthetic signal molecules. Upon addition of non-irradiated compound **3** at a concentration of 100 μM to the *P. aeruginosa* Δ*lasI* culture, we measured a modest 2-fold increase in gene expression of *lasA* (Fig. 3a). However, when compound **3** at the same concentration was irradiated with *λ* = 365 nm light prior to addition to the culture, an 18-fold increase in gene expression of *lasA* was observed, comparable (yet less strong) than the 35-fold increase in gene expression evoked by the native signalling compound **1** at 10 times lower concentration (10 μM). Compound **3** did not affect *lasA* gene expression in a *P. aeruginosa* Δ*lasRI* strain which lacks the *las* receptor and is defective in *las*-AHL production, proving that the increased gene expression is specifically induced by LasQS. Furthermore, compounds **1–3** (50 μM) did not have any substantial effect on the growth of *E. coli* JM109 pSB1075 and *P. aeruginosa* (Fig. S17 and S18†). These results show how photoswitchable QS molecules can be used to externally control the expression of (virulence) genes using light.

### Photocontrol of bacterial phenotype

Finally, we investigated the possibility to control bacterial phenotype using photoswitchable autoinducer **3**. Several phenotypes, such as biofilm formation, motility and toxin production are under the control of QS.^36^ The option to externally manipulate phenotype by employing photoswitchable QS autoinducers offers great prospect for controlling and studying bacterial group biology. One important phenotype in *P. aeruginosa* is the production of pyocyanin,^37^ which is a toxic compound that is secreted to harm competing bacteria and mammalian cells.^38^,^39^ Addition of 50 μM (2×) non-irradiated compound **3** to a *P. aeruginosa* Δ*lasI* culture resulted in only marginal pyocyanin production (Fig. 3b and S19†). However, when compound **3** was added at the same concentration, but activated by irradiation with *λ* = 365 nm light before addition, a significant increase in pyocyanin concentration (12 μM) was observed (Fig. 3b and S19†), which is in the same order of magnitude as the concentration observed for the control compound **1** (15 μM). These results indicate that it is possible to control QS-regulated phenotypes with light with the approach presented here. Future studies might focus on employing the photoswitchable autoinducers to control additional phenotypes, which may prove the usefulness of this method to externally interfere with QS and eventually use these compounds as a chemical biology tool to study bacterial group biology.

## Conclusion

We showed a proof-of-principle for using photoswitchable signalling molecules to specifically manipulate QS mechanism. A system like this, which utilizes a non-invasive and non-contaminating external stimulus, is advantageous for biological applications. QS is an attractive system that can be used as a biotechnological toolbox for synthetic biology. Exploitation of QS has been successful in the control of biofilm formation^40^ and for recombinant protein production in *E. coli.*^41^ Here, we have shown that the biosynthesis of a luminescent molecule, as well as the production of a toxic compound, can be controlled with light. In more general context, using our strategy on engineered bacterial strains, whose production of target proteins is under control of QS, would make it possible to photoregulate the protein expression. This allows the photo-control of numerous biological processes that depend on the presence and activity of selected proteins. This research paves the way for the design of efficient inducing agents with a built-in ON/OFF trigger as a tool to specifically control gene expression. Combined with the strong regulatory power of QS, an extra layer of control might be added to the circuitry of this novel chemical biology toolbox.

## Supplementary Material

Supplementary information
