# Supplementary material for: Controlling the activity of quorum sensing autoinducers with light
Source: Chem Sci. 2015 Apr 27;6(6):3593–8. doi: 10.1039/c5sc00215j (PMC5659144; doi:10.1039/c5sc00215j)
Supplement: Supplementary file 1 [file SC-006-C5SC00215J-s001.pdf]

Electronic Supplementary Material (ESI) for Chemical Science.
This journal is © The Royal Society of Chemistry 2015

Supporting Information

Title: Controlling the Activity of Quorum Sensing Autoinducers with Light

Jan Pieter van der Berg,[a§] Willem A. Velema,[b§] Wiktor Szymanski,[b,c] Arnold J. M. Driessen[a*] and Ben L. Feringa[b*]

[a]Molecular Microbiology, Groningen Biomolecular Sciences and Biotechnology Institute, Nijenborgh 7, 9747 AG, Groningen, The Netherlands

[b]Centre for Systems Chemistry, Stratingh Institute for Chemistry, University of Groningen, Nijenborgh 4, 9747 AG, Groningen, The Netherlands.

[c]Department of Radiology, University of Groningen, University Medical Centre Groningen, Groningen, The Netherlands.

[§]These authors contributed equally to the manuscript.

<u>Table of Contents</u>

Materials and Methods S2
trans:cis **ratios of compounds 2, 3 and 4** S4
UV absorption spectra of compounds 2, 3 and 4 S7
Half-life of *cis* isomer of compounds 2, 3 and 4 S8
Bioluminescence of *E. coli* reporter strain with compound 1 S10
Reversible photochromism of compounds 2, 3 and 4 S11
Growth curves of *E. coli* JM109 pSB1075 and *P. aeruginosa* in the presence of compounds 1-3 S12
Pyocyanin production S13
General synthetic scheme S14
Synthesis S14
References S18

Materials and Methods

Bacterial strains and growth conditions

Bacterial strains used in this study are *Pseudomonas aeruginosa* PA14 Δ*lasI*[1] and *E. coli* JM109 containing the plasmid pSB1075.[2] Both strains were grown in Luria Bertani (LB) broth, supplemented with 100 µg/ml gentamicin or 100 µg/ml ampicillin, respectively for *P. aeruginosa* PA14 Δ*lasI* and *E. coli* JM109 pSB1075.

Bioluminescence assay

Overnight cultures of *E. coli* JM109 pSB1075 were diluted to an optical density (OD) at 600 nm of 0.02 in LB and 100 µl of the cell suspension was added to 100 µl sterile water containing AHLs at the given concentration. To determine the effect of the AHL signaling molecules on the receptor LasR after photo-irradiation, stock solutions in DMSO were first irradiated at λ=365 nm for 10 minutes, after which the solutions were diluted in LB broth to the right concentration, before adding the cell suspension. To measure multiple rounds of switching between *cis*- and *trans*-isomers, the solutions were sequentially photo-irradiated at λ=365 nm for 10 min using a Spectroline ENB-280C/FE UV lamp, followed by white light for 30 min using a Thor Labs OSL1-EC Fiber Illuminator. Next, the samples were irradiated for a second time at λ=365 nm for 10 min. The cell suspensions with or without AHL compounds were incubated for 2-3 hours at 30 °C in a white microtiter plate and the luminescence was measured at 10 minute intervals using a microtiter plate luminometer (Orion L, Berthold detection systems). The maximum luminescence, occurring after approximately 1.5 hours, was used to compare the AHL effect on *las* activity. Measurements were performed on two biological replicates, each performed in triplo. The normalized average and pooled variance (calculated using equation 1) of the maximum luminescence were plotted for each AHL concentration.

Equation 1: $$s_p^2 = \frac{\sum_{i=1}^{k}(n_i - 1)s_i^2}{\sum_{i=1}^{k}(n_i - 1)}$$

RNA isolation and cDNA synthesis

Overnight cultures of *P. aeruginosa* PA14 Δ*lasI* were 100-fold diluted in a fresh LB medium and grown to an OD_{600} of 2.5 at 37 °C. The preculture was diluted to an OD_{600} of 0.5 and subsequently the AHLs in DMSO were added to result in the given concentrations. As a control, an equal volume of DMSO was used. After 90 minutes of growth, 5 OD units of each culture was centrifuged for 15 minutes, 3000 x g at 4 °C. The resulted pellet was resuspended in MilliQ water and total RNA was isolated using TRIzol (Life Technologies), followed by DNAse treatment using the Turbo DNA-free kit (Ambion). The total RNA concentration was measured using a Nanodrop ND-1000; 500 ng of total RNA was used to synthesize cDNA using iScript cDNA synthesis kit (Bio-Rad).

Gene expression with qPCR

The expression of the gene of interest, *lasA*, and the reference gene *rpoD* were analyzed using qPCR with the primers listed in table S1. A negative control reaction with total RNA was analyzed to exclude gDNA contamination. As a PCR mastermix, the SYBR mix (Bioline) was used with 0.4 μM primers. Expression levels of the genes of interest of two biological replicates, each performed in duplo, were measured with a MiniOpticon system (Bio-Rad) and results were corrected for the control sample and normalized against the reference gene.

Table S1 Primers used for gene expression analysis

**Primer**	**Sequence 5'-3'**	**Reference**
lasA F	TCCTTCGATGCGTCCTAC	This work
lasA R	GTTGCTCACCTGGATCTG	This work
rpoD F	TTCCTCGTCGTCCTTCTC	This work
rpoD R	TCCTGGCCGACTACAATC	This work

Pyocyanin assay

Overnight cultures of *Pseudomonas aeruginosa* PA14 □*lasI* grown in peptone broth (20 g/l Bacto Peptone, 10 g/l K_2SO_4, 1.5 g/l $MgSO_4$) were diluted to an OD_{600} of 1.0 and grown for 30 minutes at 37 °C. Subsequently, 50 μM of compound **1** or compound **3**, non-irradiated or λ=365 nm light-irradiated were added to the culture and incubated for 2 at 37 °C. Then a second dose of 50 μM compound **3,** non-irradiated or λ=365 nm light-irradiated, was added and the cultures were incubated for another 3 h at 37 °C.

The pyocyanin concentration was determined according to a previously published method.[3] In short, after incubation the cultures were centrifuged, supernatant was filtered using a 0.2 μm filter and the pyocyanin production was determined by measuring the absorbance at 690 nm using an Agilent 8453 UV-Visible Spectrophotometer. The absorbance was converted to pyocyanin concentration using the extinction coefficient of 4310 M^{-1} cm^{-1}. The results were background-corrected by subtracting the signal obtained from a control that was incubated with an equal volume of DMSO only.

Photoswitching Experiments Irradiation experiments were performed with a Spectroline ENB-280C/FE UV lamp (365 nm) and Thor Labs OSL1-EC Fiber Illuminator (white light).

Synthesis *General.* All chemicals for synthesis were obtained from commercial sources and were used as received, unless stated otherwise. Solvents were reagent grade. Thin-layer chromatography (TLC) was performed using commercial Kieselgel 60, F254 silica gel plates. Flash chromatography was performed on silica gel (Silicycle Siliaflash P60, 40-63 μm, 230-400 mesh). Drying of solutions was performed with $MgSO_4$ and solvents were removed with a rotary evaporator. Chemical shifts for NMR measurements were determined relative to the residual solvent peaks (δ_H 7.26 for $CHCl_3$ and 2.50 for DMSO, δ_C 77.16 for $CDCl_3$ and 39.52 for DMSO). The following abbreviations are used to indicate signal multiplicity: s, singlet; d, doublet; t, triplet; q, quartet; m, multiplet; brs, broad signal; appt, apparent triplet. HRMS (ESI) spectra were obtained on a Thermo scientific LTQ Orbitrap XL. Melting points were recorded using a Buchi melting point B-545 apparatus. UV/Vis absorption spectra were recorded on an Agilent 8453 UV-Visible Spectrophotometer using Uvasol grade solvents.

Trans:cis ratios of compound 2, 3 and 4

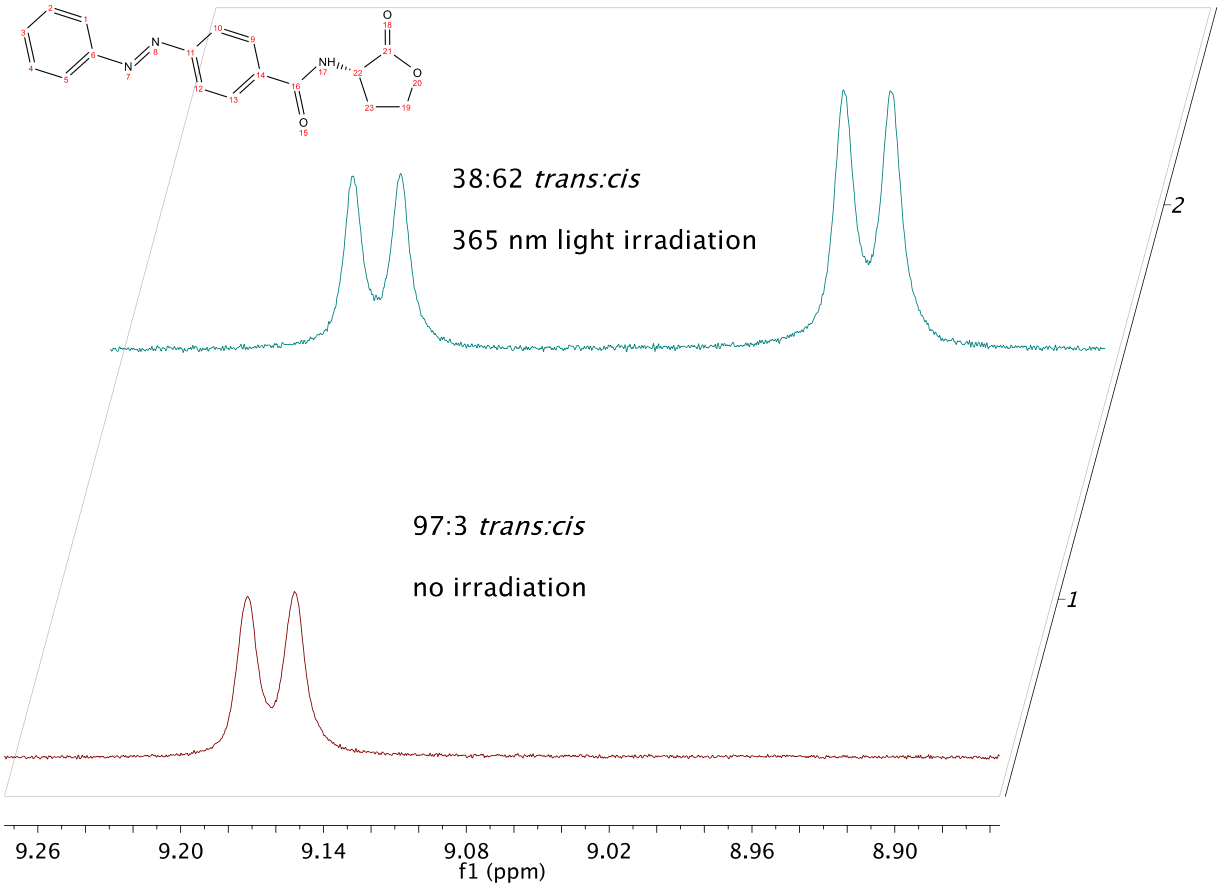

Figure S1 *Trans:cis* ratio of compound **2** at a concentration of 10 mM in DMSO-d_6 before (bottom) and after 365 nm light irradiation (top), determined by integration of the N-H (nr. 17 in molecular structure) signal of compound **2** using ^{1}H NMR spectroscopy.

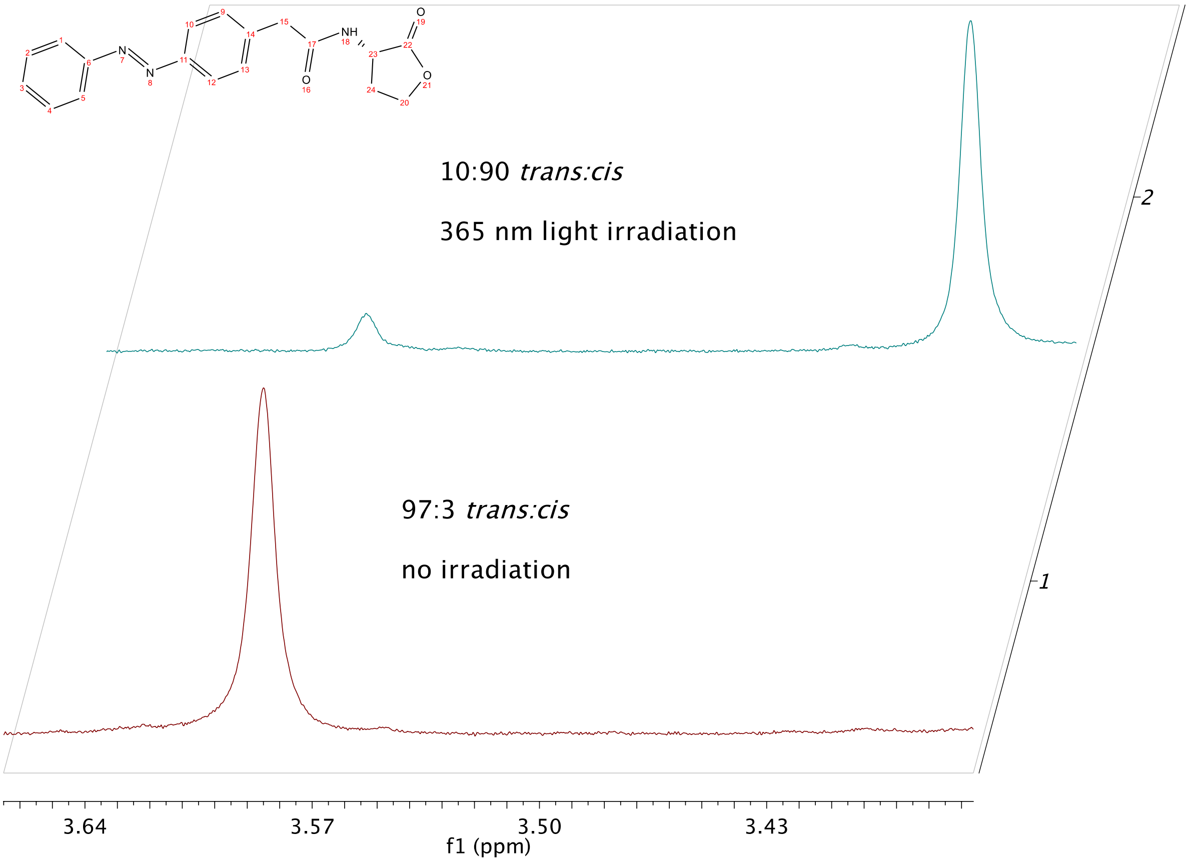

Figure S2 *Trans:cis* ratio of compound **3** at a concentration of 10 mM in DMSO-d_6 before (bottom) and after 365 nm light irradiation (top), determined by integration of the CH_2 signal (nr. 15 in molecular structure) of compound **3** using ^{1}H NMR spectroscopy.

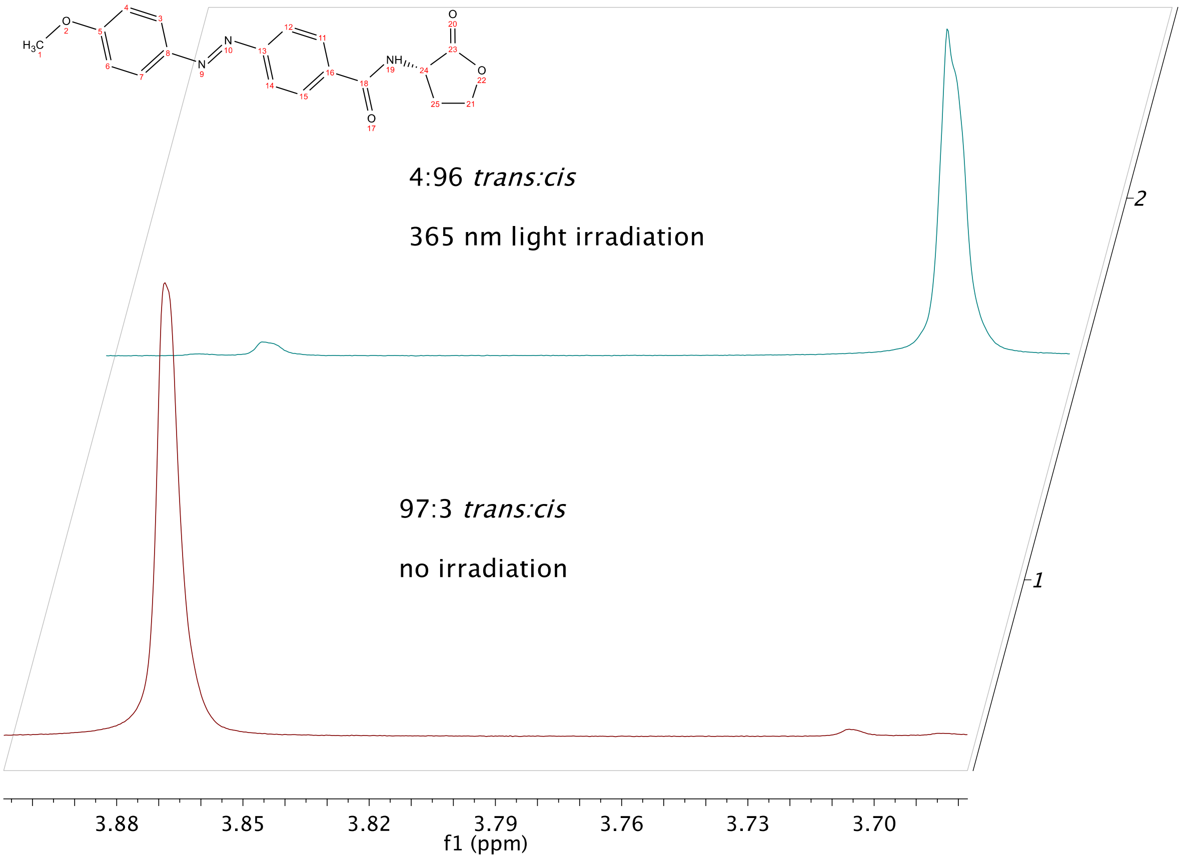

Figure S3 *Trans:cis* ratio of compound **4** at a concentration of 10 mM in DMSO-d_6 before (bottom) and after 365 nm light irradiation (top), determined by integration of the CH_3 signal (nr. 1 in molecular structure) of compound **4** using ^1H NMR spectroscopy.

UV-vis absorption spectra of compound 2, 3 and 4

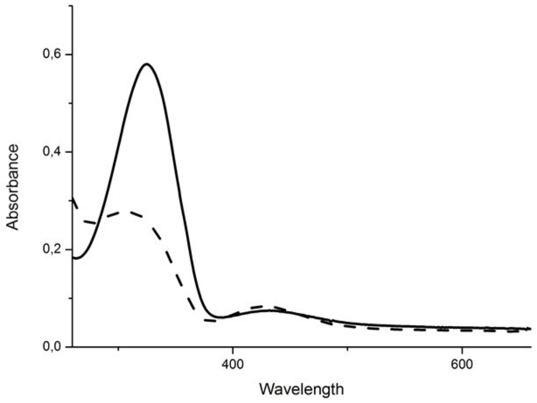

Figure S4 UV-Vis absorption spectra of compound **2** at a concentration of 50 µM in DMSO. *Trans* isomer is solid line, *cis* isomer is dashed line.

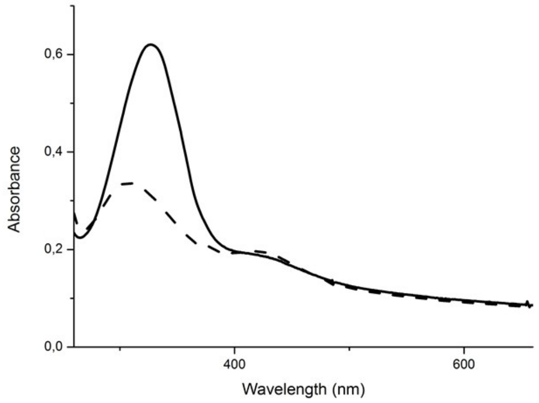

Figure S5 UV-Vis absorption spectra of compound **3** at a concentration of 50 µM in DMSO. *Trans* isomer is solid line, *cis* isomer is dashed line.

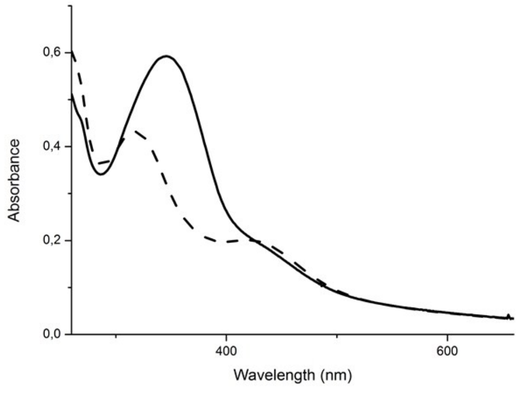

Figure S6 UV-Vis absorption spectra of compound **4** at a concentration of 50 µM in DMSO. *Trans* isomer is solid line, *cis* isomer is dashed line.

Half-lives of compound 2, 3 and 4 at 30 °C and 37 °C

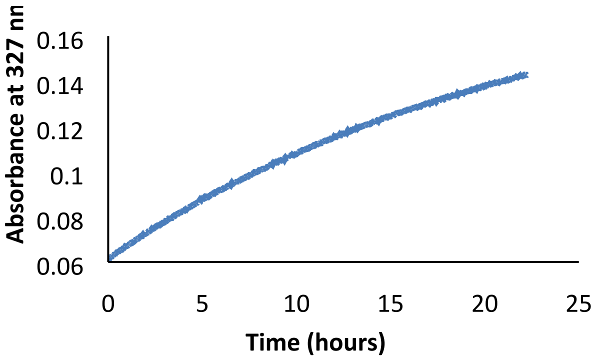

Figure S7 Changes in absorption at λ = 327 nm of the *cis*-isomer of compound **2** at a concentration of 11 μM in water at 30 °C. Half-life > 10 hours.

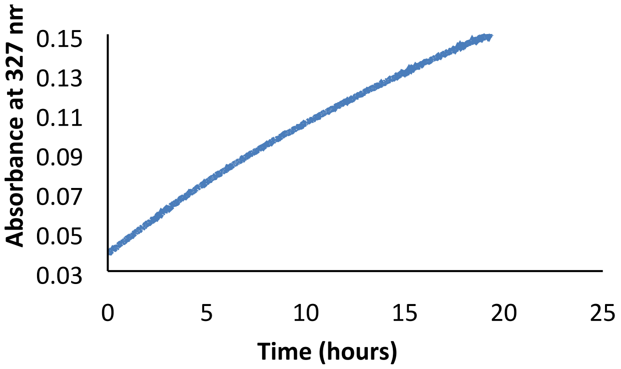

Figure S8 Changes in absorption at λ = 327 nm of the *cis*-isomer of compound **2** at a concentration of 7.1 μM in water at 37 °C. Half-life > 10 hours.

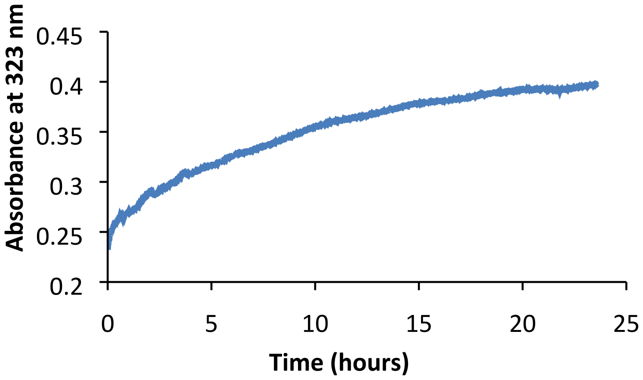

Figure S9 Changes in absorption at λ = 323 nm of the *cis*-isomer of compound **3** at a concentration of 38 μM in water at 30 °C. Half-life is 6.8 hours.

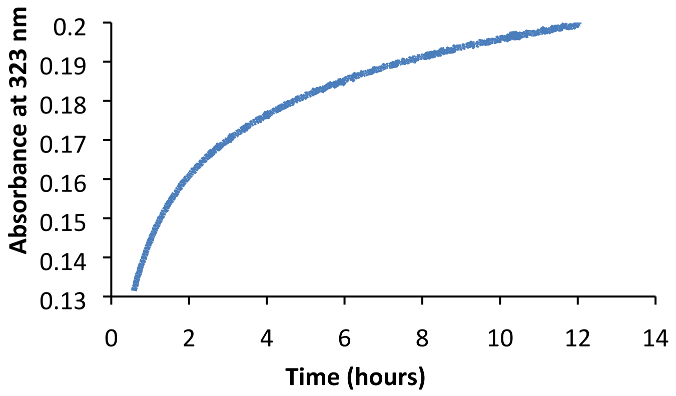

Figure S10 Changes in absorption at λ = 323 nm of the *cis*-isomer of compound **3** at a concentration of 19 μM in water at 37 °C. Half-life is 2.5 hours.

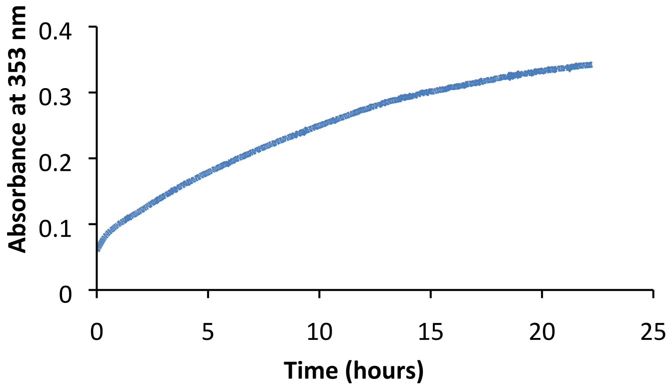

Figure S11 Changes in absorption at λ = 353 nm of the *cis*-isomer of compound **4** at a concentration of 8.6 μM in water at 30 °C. Half-life is 8.9 hours.

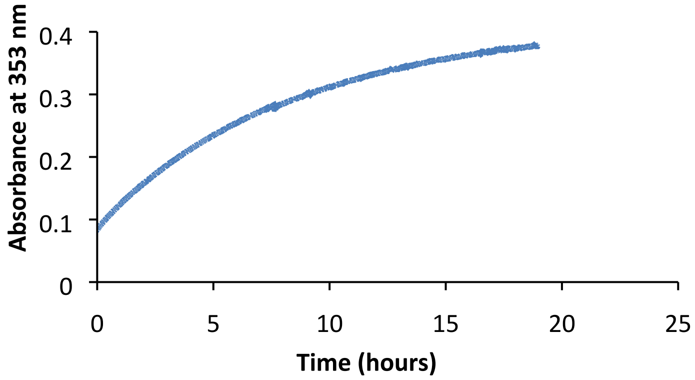

Figure S12 Changes in absorption at λ = 353 nm of the *cis*-isomer of compound **4** at a concentration of 11.4 μM in water at 37 °C. Half-life is 5.8 hours.

Bioluminescence dose-response curves of compound 1

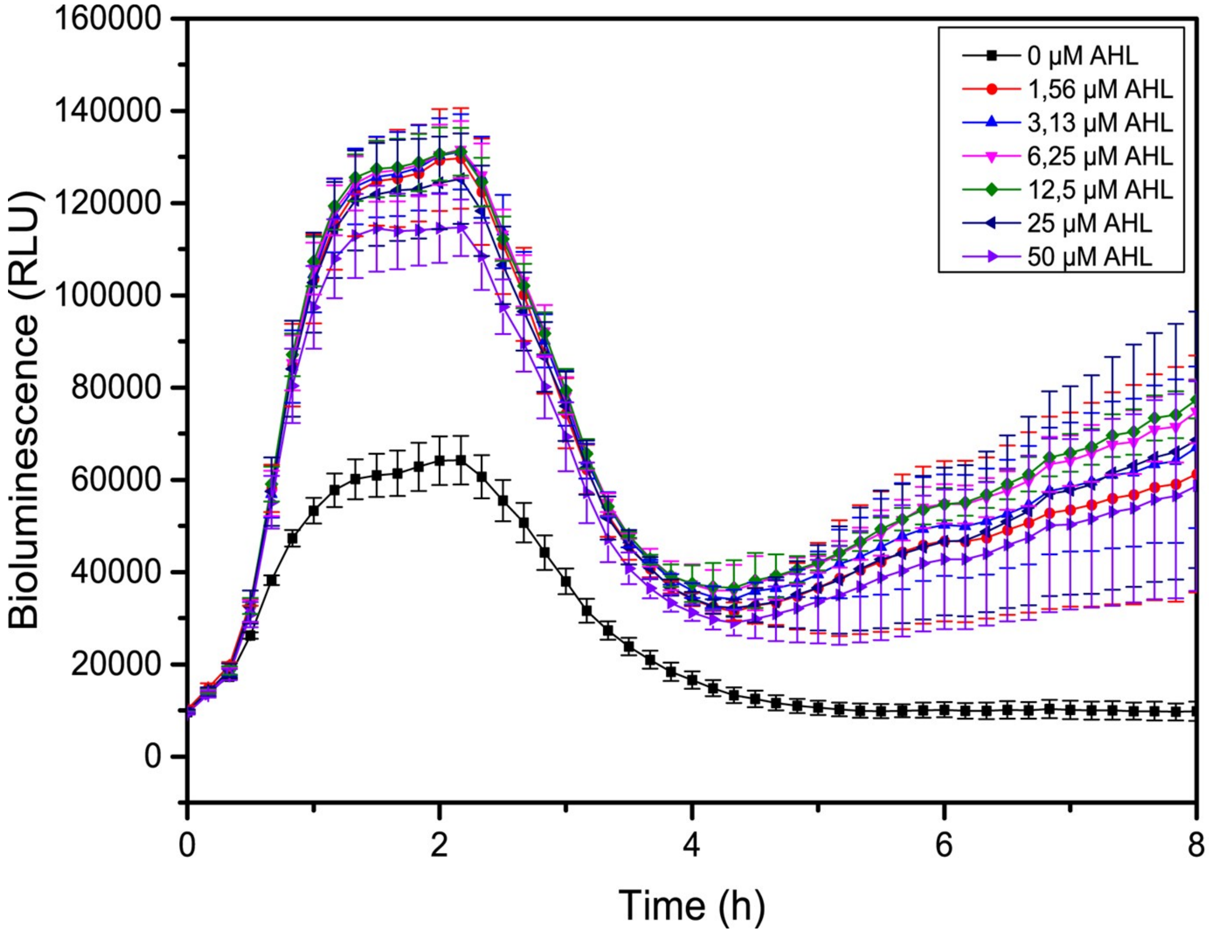

Figure S13 Bioluminescence observed in the *E. coli* sensor strain over time, after addition of different concentrations of compound **1.**

Reversible photochromism of compounds 2, 3 and 4

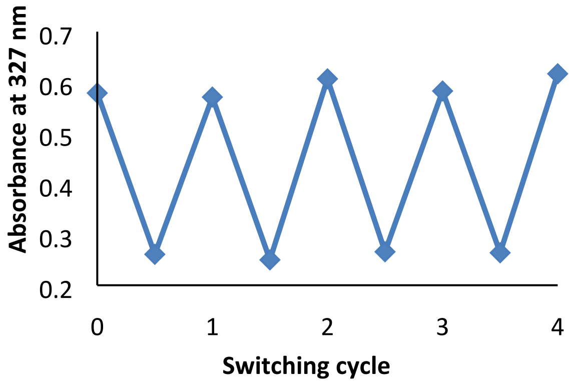

Figure S14 Reversible photochromism of compound **2** at a concentration of 50 µM in DMSO.

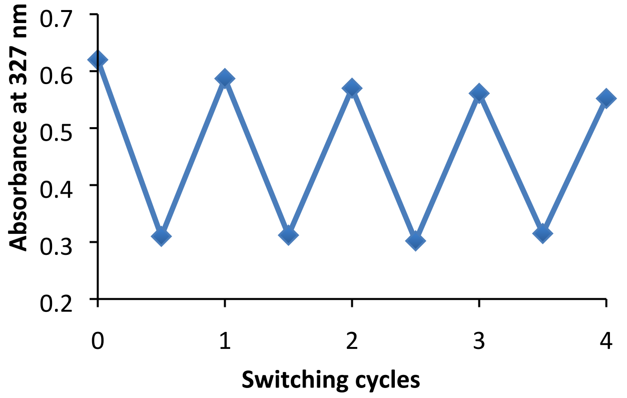

Figure S15 Reversible photochromism of compound **3** at a concentration of 50 µM in DMSO.

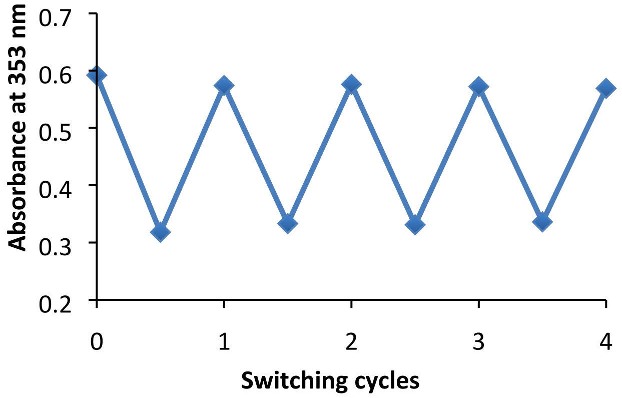

Figure S16 Reversible photochromism of compound **4** at a concentration of 50 µM in DMSO

Growth curves of *E. coli* JM109 pSB1075 and *P. aeruginosa* in the presence of compounds 1-3

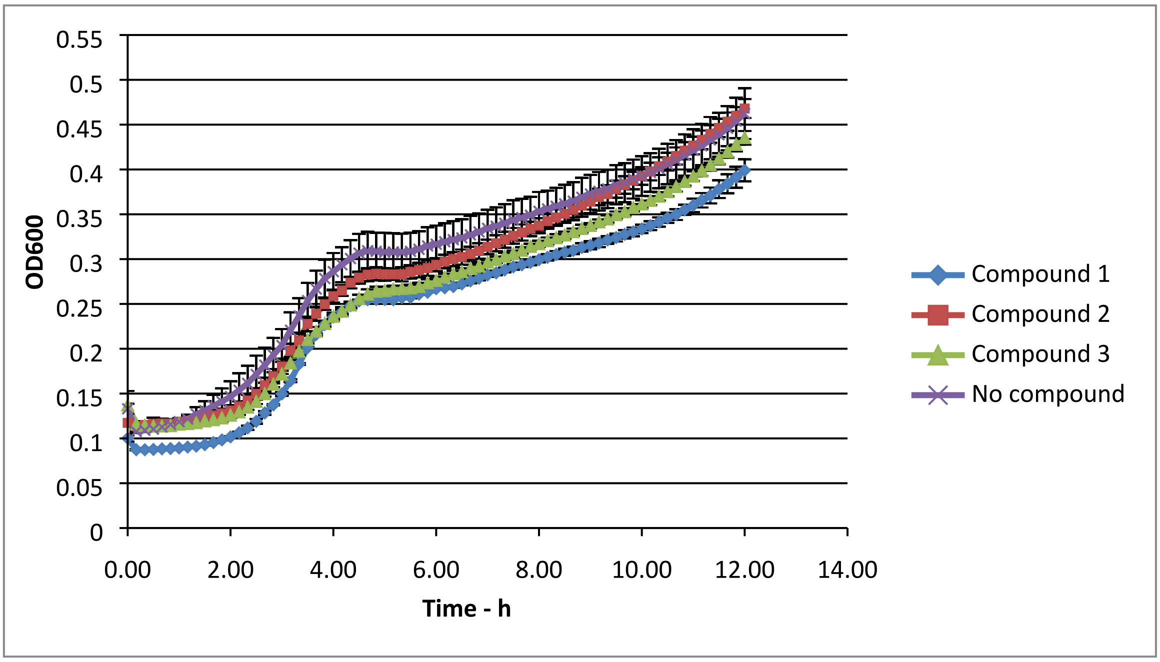

Figure S17 Growth curve of *E. coli* JM109 pSB1075 in the presence of 50 µm of compound **1-3**.

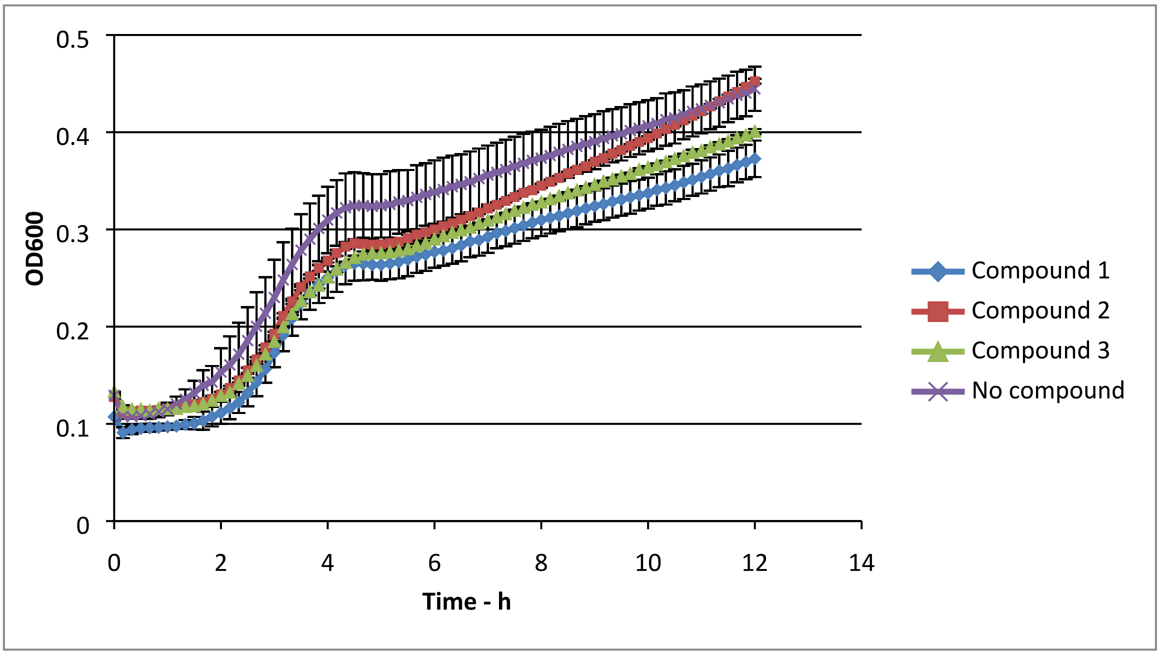

Figure S18 Growth curve of *P. aeruginosa* in the presence of 50 µm of compound **1-3**.

Pyocyanin Production

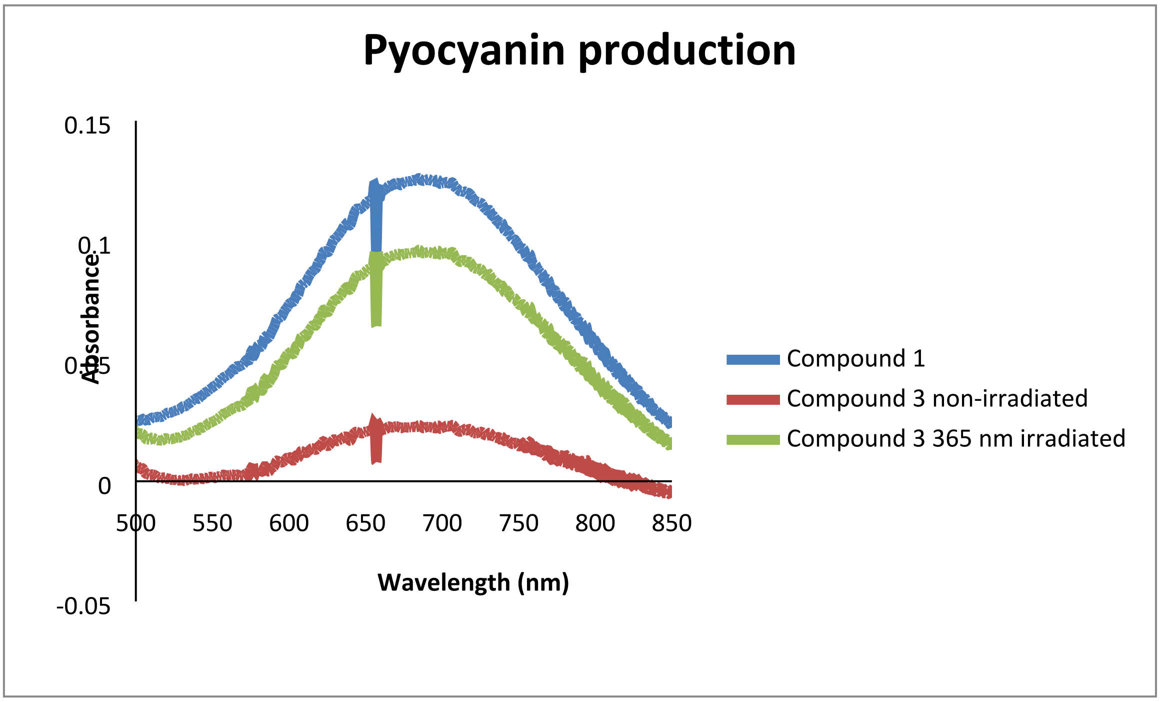

Figure S19 UV-Vis absorption spectra of filtered supernatant of *P. aeruginosa* Δ*lasI* cultures incubated with compound **1** (50 µM), compound **3** (2x50 µM), and compound **3** (2x50 µM), exposed to 365 nm light.

General synthetic scheme

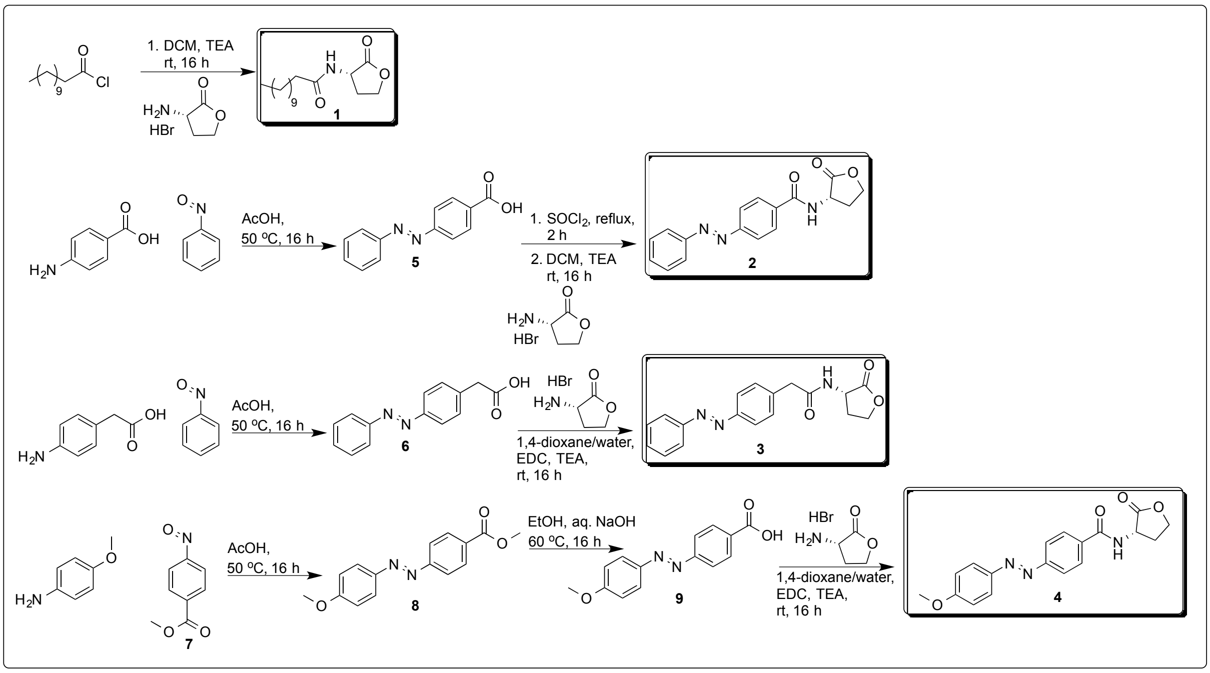

Synthesis

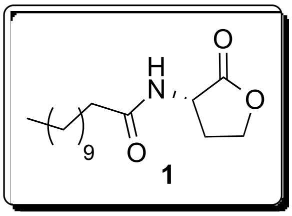

(S)-N-(2-oxotetrahydrofuran-3-yl)butyramide (**1**) Lauroyl chloride (0.46 mmol, 100 mg) was dissolved in DCM (5 mL). (*S*)-(−)-α-Amino-γ-butyrolactone hydrobromide (0.46 mmol, 84 mg) was added, followed by triethylamine (1.38 mmol, 140 mg), and the resulting solution was stirred for 16 h at room temperature. Next, the solution was concentrated *in vacuo* and the residue was dissolved in EtOAc (20 mL). This solution was washed with 1 M aq. HCl (20 mL) and brine (20 mL) and dried ($MgSO_4$). Removing the solvent *in vacuo* resulted in 110 mg (85%) of a white solid. Mp. 140-142 °C.

^{1}H NMR (400 MHz, $CDCl_3$): δ 5.93 (s, 1H), 4.61 - 4.42 (m, 2H), 4.28 (ddd, *J* = 11.3, 9.3, 5.8 Hz, 1H), 2.95 - 2.83 (m, 1H), 2.28 - 2.20 (m, 2H), 2.11 (ddd, *J* = 23.9, 11.6, 8.8 Hz, 1H), 1.69 – 1.62 (m, 2H), 1.38 – 1.24 (m, 16H), 0.88 (t, *J* = 6.8 Hz, 3H).
^{1}H NMR spectrum in agreement with published data.[4]

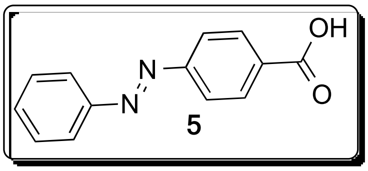

(E)-4-(phenyldiazenyl)benzoic acid (**5**) Nitrosobenzene (1.86 mmol, 200 mg) and 4-aminobenzoic acid (2.05 mmol, 282 mg) were dissolved in acetic acid (15 mL) and the mixture was stirred for 16 h at 60 °C. Next, the solution was diluted with water (30 mL) and extracted with DCM (30 mL). The organic layer was washed with water (30 mL) and brine (30 mL) and dried ($MgSO_4$). After concentrating *in vacuo*, the solid was recrystallized from EtOAc, resulting in 360 mg (85%) of an orange solid.

^{1}H NMR (400 MHz, DMSO-d$_6$): δ 13.22 (brs, 1H), 8.13 (d, *J* = 8.4 Hz, 2H), 7.98 – 7.88 (m, 4H), 7.65 – 7.56 (m, 3H).
^{13}C NMR (100 MHz, DMSO-d$_6$): δ 167.1, 154.7, 152.3, 133.3, 132.6, 131.1, 130.0, 123.3, 123.0.
^{1}H NMR spectrum in agreement with published data.[5]

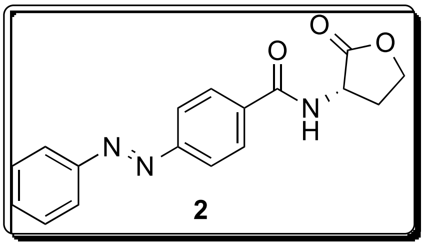

2

(S)-N-(2-oxotetrahydrofuran-3-yl)-4-(phenyldiazenyl)benzamide (**2**) Compound **5** (0.42 mmol, 95 mg) was dissolved in thionyl chloride (2 mL) and the mixture was heated at reflux for 2 h. The mixture was concentrated *in vacuo* and the residue was redissolved in DCM. To the solution was added (*S*)-(−)-α-amino-γ-butyrolactone hydrobromide (0.42 mmol, 76 mg) and triethylamine (1.26 mmol, 127 mg) and the mixture was stirred for 16 h at room temperature. Next, the solution was concentrated *in vacuo* and the residue was dissolved in EtOAc (20 mL). The organic solution was washed with 1 M HCl (20 mL) and brine (20 mL) and dried ($MgSO_4$). Concentrating *in vacuo* resulted in 120 mg (93%) of an orange solid. Mp. 229-231 °C.

^{1}H NMR (400 MHz, DMSO-d$_6$): δ 9.16 (d, *J* = 8.2 Hz, 1H), 8.06 (d, *J* = 8.5 Hz, 2H), 7.97 (d, *J* = 8.5 Hz, 2H), 7.92 (dd, *J* = 7.6, 2.0 Hz, 2H), 7.66 – 7.58 (m, 3H), 4.81 (apq, *J* = 9.2 Hz, 1H), 4.43 (apt, *J* = 8.1 Hz, 1H), 4.32 – 4.24 (m, 1H), 2.43 – 2.28 (m, 2H).
^{13}C NMR (100 MHz, DMSO-d$_6$): δ175.6, 165.7, 153.9, 152.4, 136.0, 132.5, 130.0, 129.1, 123.2, 122.9, 65.8, 49.0, 28.4.
HR-MS (ESI, [M+H]$^+$): Calcd. for $C_{17}H_{16}N_3O_3$: 310.1186; Found: 310.1186

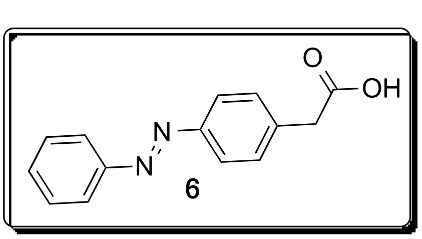

6

(E)-2-(4-(phenyldiazenyl)phenyl)acetic acid (**6**) Nitrosobenzene (1.86 mmol, 200 mg) and 4-aminophenylacetic acid (2.05 mmol, 310 mg) were dissolved in acetic acid (15 mL) and the solution was stirred for 16 h at 50 °C. Next, the solution was diluted with water (30 mL) and

extracted with DCM (30 mL). The organic layer was washed with water (30 mL) and brine (30 mL) and dried ($MgSO_4$). After concentrating *in vacuo*, the solid was recrystallized from EtOAc, resulting in 400 mg (89%) of an orange solid.

^{1}H NMR (400 MHz, DMSO-d$_6$): δ 12.44 (brs, 1H), 7.89 – 7.81 (m, 4H), 7.62 – 7.54 (m, 3H), 7.47 (d, *J* = 8.3 Hz, 2H), 3.69 (s, 2H).
^{13}C NMR (100 MHz, DMSO-d$_6$): δ 172.7, 152.4, 151.2, 139.3, 131.9, 131.0, 129.9, 122.9, 122.9, 40.8.
^{1}H NMR spectrum in agreement with published data.[6]

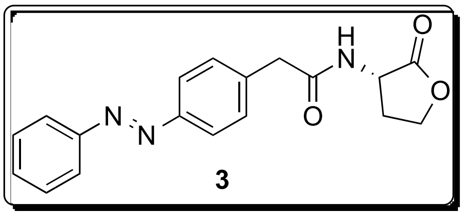

3

(S)-N-(2-oxotetrahydrofuran-3-yl)-2-(4-(phenyldiazenyl)phenyl)acetamide (**3**) Compound **6** (0.42 mmol, 100 mg) was dissolved in 1,4-dioxane (4 mL) and added to a solution of (*S*)-(−)-α-amino-γ-butyrolactone hydrobromide (0.42 mmol, 76 mg) in water (2 mL). Next, TEA (1.25 mmol, 126 mg) and EDC hydrochloride (0.63 mmol, 120 mg) were added, and the reaction mixture was stirred for 16 h at room temperature. The mixture was added to EtOAc (20 mL) and washed with a 5% aqueous citric acid solution (20 mL) and brine (20 mL) and dried ($MgSO_4$). Concentrating *in vacuo* resulted in 55 mg (40%) of an orange solid. Mp. 222-224 ^{o}C.

^{1}H NMR (400 MHz, DMSO-d$_6$): δ 8.66 (d, *J* = 7.7 Hz, 1H), 7.85 (dd, *J* = 13.5, 7.5 Hz, 4H), 7.62 – 7.53 (m, 3H), 7.47 (d, *J* = 8.2 Hz, 2H), 4.57 (apq, *J* = 8.8 Hz, 1H), 4.33 (t, *J* = 8.1 Hz, 1H), 4.24 – 4.15 (m, 1H), 3.59 (s, 1H), 2.40 (dd, *J* = 17.6, 8.9 Hz, 1H), 2.20 – 2.07 (m, 1H).
^{13}C NMR (100 MHz, DMSO-d$_6$): δ 175.7, 170.2, 152.4, 151.1, 140.2, 131.9, 130.6, 129.9, 122.9, 122.9, 65.7, 48.6, 42.2, 28.7.
HR-MS (ESI, [M+H]$^+$): Calcd. for $C_{18}H_{18}N_3O_3$: 324.1343; Found: 324.1341

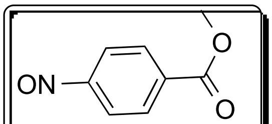

7

Methyl 4-nitrosobenzoate (**7**) Methyl-4-aminobenzoate (3.30 mmol, 500 mg) was dissolved in DCM (10 mL) and a solution of oxone (6.60 mmol, 4.10 g) in water (50 mL) was added. The resulting biphasic mixture was stirred at room temperature for 3 h. Next, the organic layer was separated and the aqueous layer was extracted with DCM (2 x 20 mL). The combined organic layers were washed with 1M aqueous HCl (30 mL) and brine (20 mL) and dried ($MgSO_4$). Drying *in vacuo* and recrystallization from ethyl acetate resulted in 431 mg (79%) of a bright yellow solid.

^{1}**H NMR (400 MHz, $CDCl_3$)**: δ 8.30 (d, *J* = 8.6 Hz, 2H), 7.94 (d, *J* = 8.6 Hz, 2H), 3.98 (s, 3H).
^{1}H NMR spectrum in agreement with published data.[7]

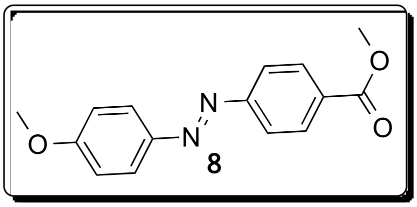

Methyl (E)-4-((4-methoxyphenyl)diazenyl)benzoate (**8**)
p-Anisidine (0.66 mmol, 81 mg) and compound **7** (0.60 mmol, 100 mg) were dissolved in acetic acid (5 mL) and the resulting solution was stirred at 40 °C for 16 h. Next, the solution was diluted with water (40 mL) and extracted with DCM (3 x 40 mL). The collected organic phases were dried ($MgSO_4$). The solvent was evaporated *in vacuo*, resulting in 159 mg (>95%) of orange crystals.
^{1}**H NMR (400 MHz, $CDCl_3$):** δ 8.17 (d, *J* = 8.6 Hz, 2H), 7.96 (d, *J* = 9.0 Hz, 2H), 7.91 (d, *J* = 8.6 Hz, 2H), 7.03 (d, *J* = 9.0 Hz, 2H), 3.96 (s, 3H), 3.91 (s, 3H).
^{1}H NMR spectrum in agreement with published data.[8]

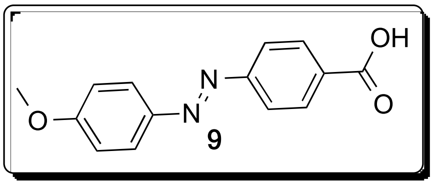

(E)-4-((4-methoxyphenyl)diazenyl)benzoic acid (**9**) Compound **8** (0.660 mmol, 180 mg) was added to a solution of 2.5 M aqueous NaOH (1 mL) in EtOH (15 mL) and the mixture was stirred at 60 °C for 16 h. Subsequently, 1M aqueous HCl (30 mL) was added and the mixture was extracted with DCM (3 x 20 mL). Removing the solvent *in vacuo* resulted in 161 mg (>95%) of an orange solid.
^{1}**H NMR (400 MHz, DMSO-d_6):** δ 8.10 (d, *J* = 8.6 Hz, 2H), 7.93 (d, *J* = 9.0 Hz, 2H), 7.90 (d, *J* = 8.6 Hz, 2H), 7.15 (d, *J* = 9.1 Hz, 2H), 3.87 (s, 3H).
^{1}H NMR spectrum in agreement with published data.[9]

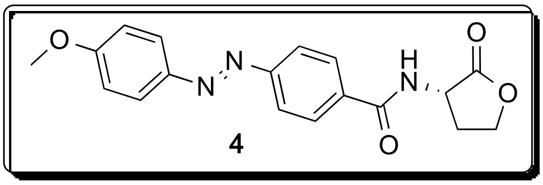

(S)-N-(2-oxotetrahydrofuran-3-yl)-2-(4-(phenyldiazenyl)phenyl)acetamide (**4**) Compound **9** (0.39 mmol, 100 mg) was dissolved in 1,4-dioxane (4 mL) and added to a solution of (*S*)-(−)-α-amino-γ-butyrolactone hydrobromide (0.39 mmol, 71 mg) in water (1 mL). Next, triethylamine (0.78 mmol, 118 mg) and EDC hydrochloride (0.59 mmol, 111 mg) were added, and this solution was stirred for 16 h at room temperature. The mixture was diluted with EtOAc (20 mL) and washed with a 5% aqueous citric acid solution (20 mL) and brine (20

mL) and dried ($MgSO_4$). Concentrating *in vacuo* resulted in 50 mg (30%) of an orange solid. Mp. 210-212 ^oC.

^{1}H NMR (400 MHz, DMSO-d$_6$): δ 9.13 (d, J = 7.5 Hz, 1H), 8.12 – 8.02 (m, 2H), 7.96 – 7.87 (m, 4H), 7.15 (d, J = 8.7 Hz, 2H), 4.80 (apq, J = 9.3 Hz, 1H), 4.42 (apt, J = 8.6 Hz, 1H), 4.28 (apq, J = 9.0 Hz, 1H), 3.87 (s, 3H), 2.36 (m, 2H).
^{13}C NMR (100 MHz, DMSO-d$_6$): δ 175.7, 165.8, 162.9, 154.1, 146.7, 135.3, 131.0, 129.0, 125.4, 125.4, 125.4, 122.6, 115.2, 65.8, 56.2, 49.0, 28.4.
HR-MS (ESI, [M+H]$^+$): Calcd. for $C_{18}H_{18}N_3O_4$: 340.1292; Found: 340.1291

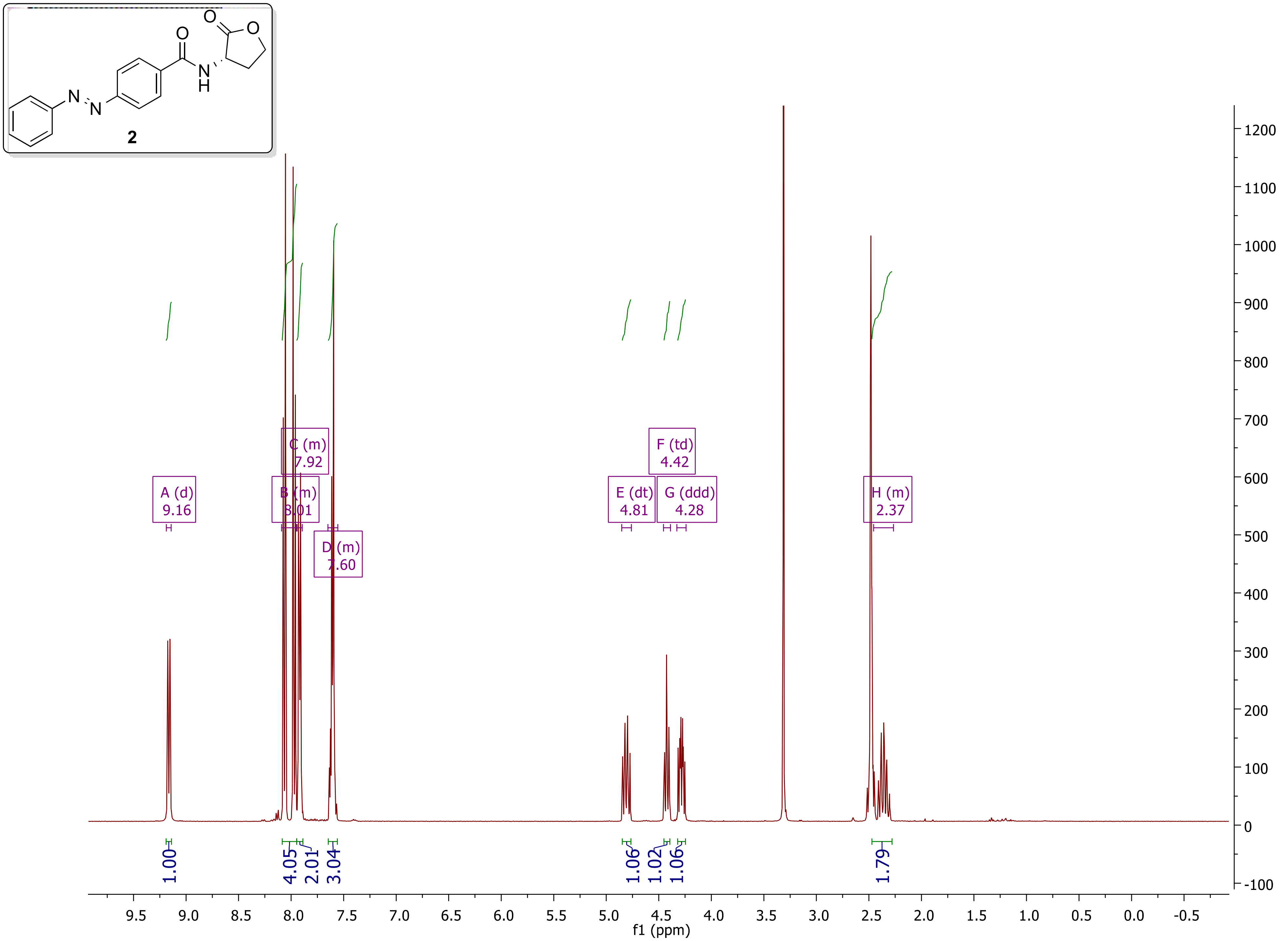

2
A (d)
9.16
B (m)
8.01
C (m)
7.92
D (m)
7.60
E (dt)
4.81
F (td)
4.42
G (ddd)
4.28
H (m)
2.37
1.00
4.05
2.01
3.04
1.06
1.02
1.06
1.79
f1 (ppm)

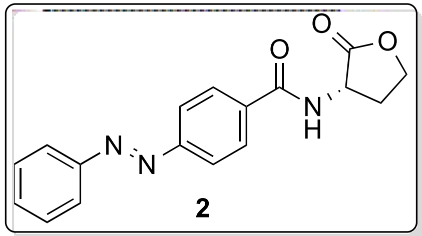

2

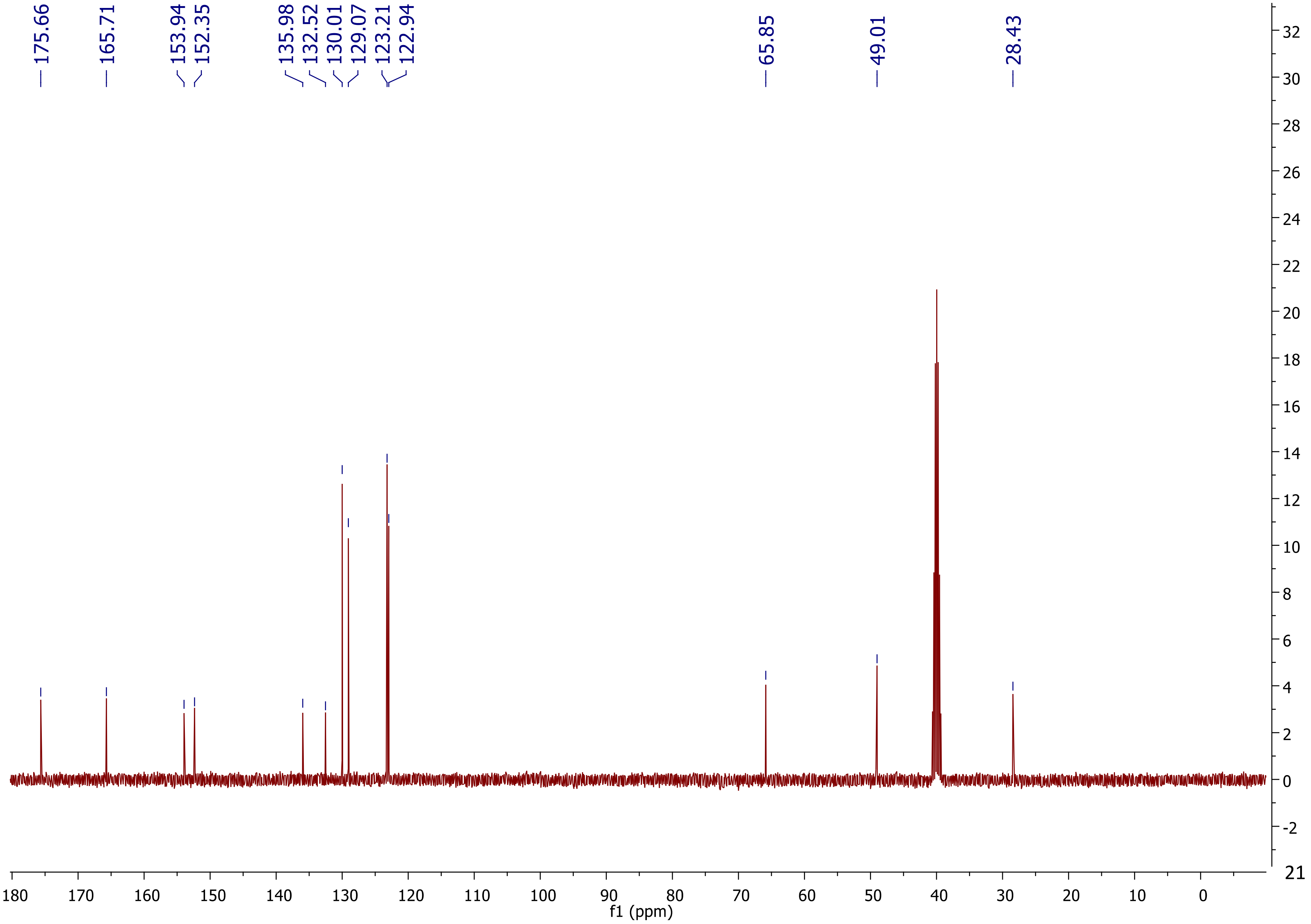
175.66
165.71
153.94
152.35
135.98
132.52
130.01
129.07
123.21
122.94
65.85
49.01
28.43
180 170 160 150 140 130 120 110 100 90 80 70 60 50 40 30 20 10 0
f1 (ppm)
32 30 28 26 24 22 20 18 16 14 12 10 8 6 4 2 0 -2

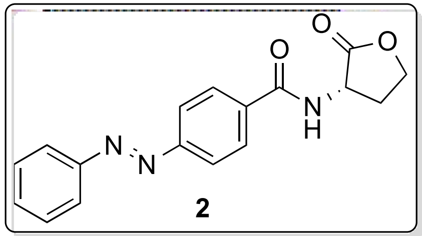

2

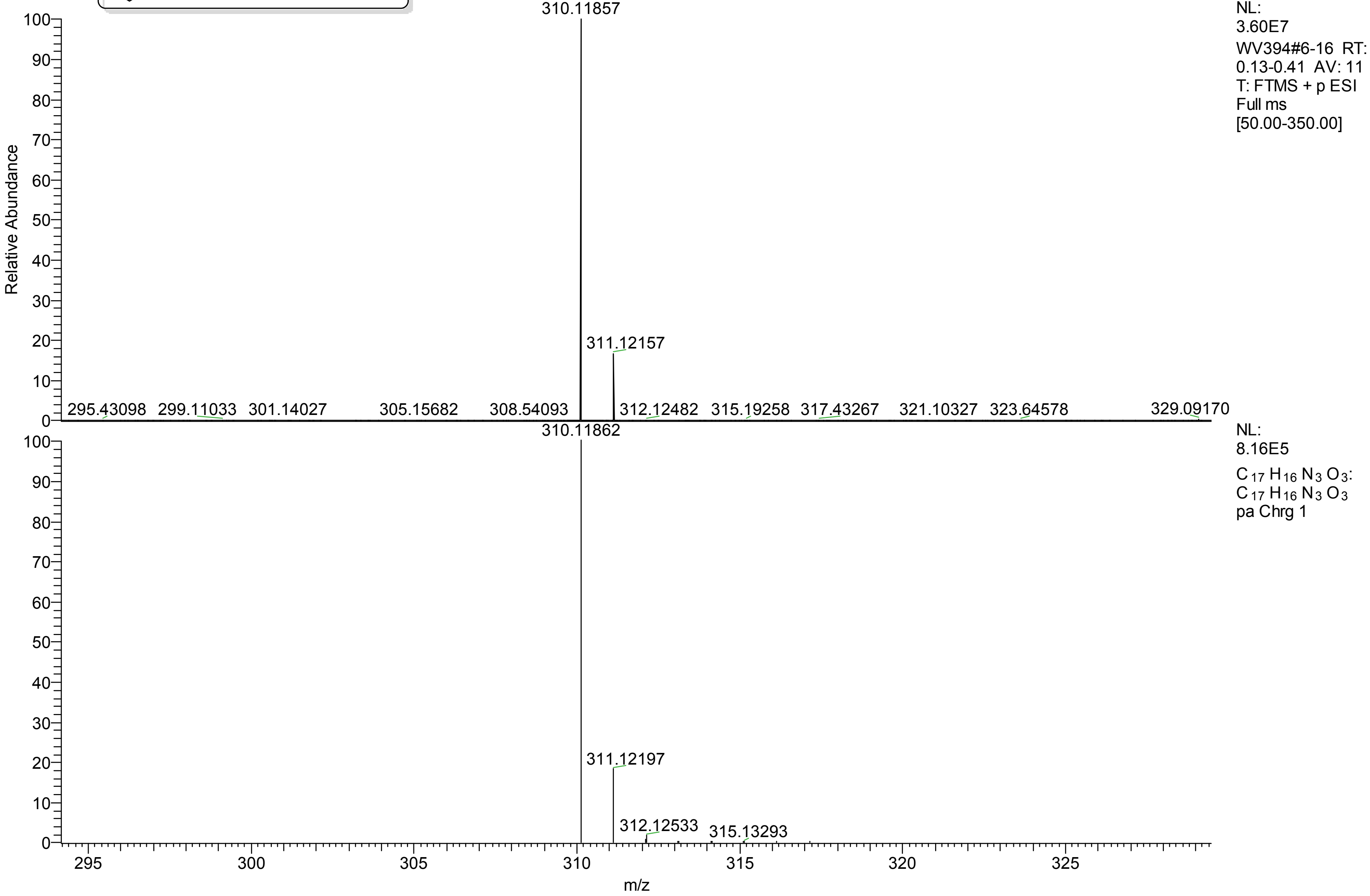

310.11857
NL:
3.60E7
WV394#6-16 RT:
0.13-0.41 AV: 11
T: FTMS + p ESI
Full ms
[50.00-350.00]
Relative Abundance
311.12157
295.43098
299.11033
301.14027
305.15682
308.54093
312.12482
315.19258
317.43267
321.10327
323.64578
329.09170
310.11862
NL:
8.16E5
C 17 H16 N3 O3:
C 17 H16 N3 O3
pa Chrg 1
311.12197
312.12533
315.13293
m/z

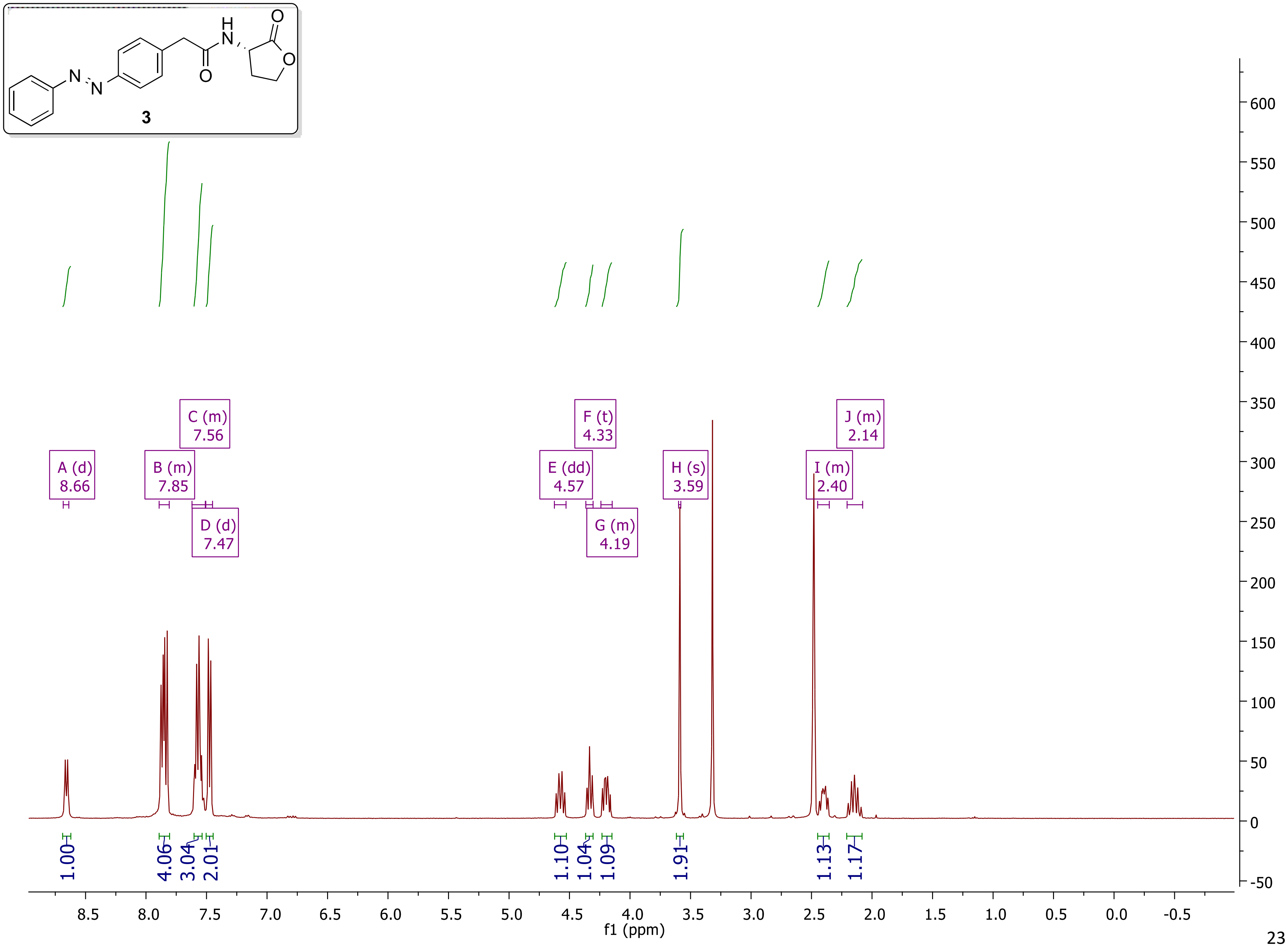

3
A (d) 8.66
B (m) 7.85
C (m) 7.56
D (d) 7.47
E (dd) 4.57
F (t) 4.33
G (m) 4.19
H (s) 3.59
I (m) 2.40
J (m) 2.14
1.00
4.06
3.04
2.01
1.10
1.04
1.09
1.91
1.13
1.17
f1 (ppm)

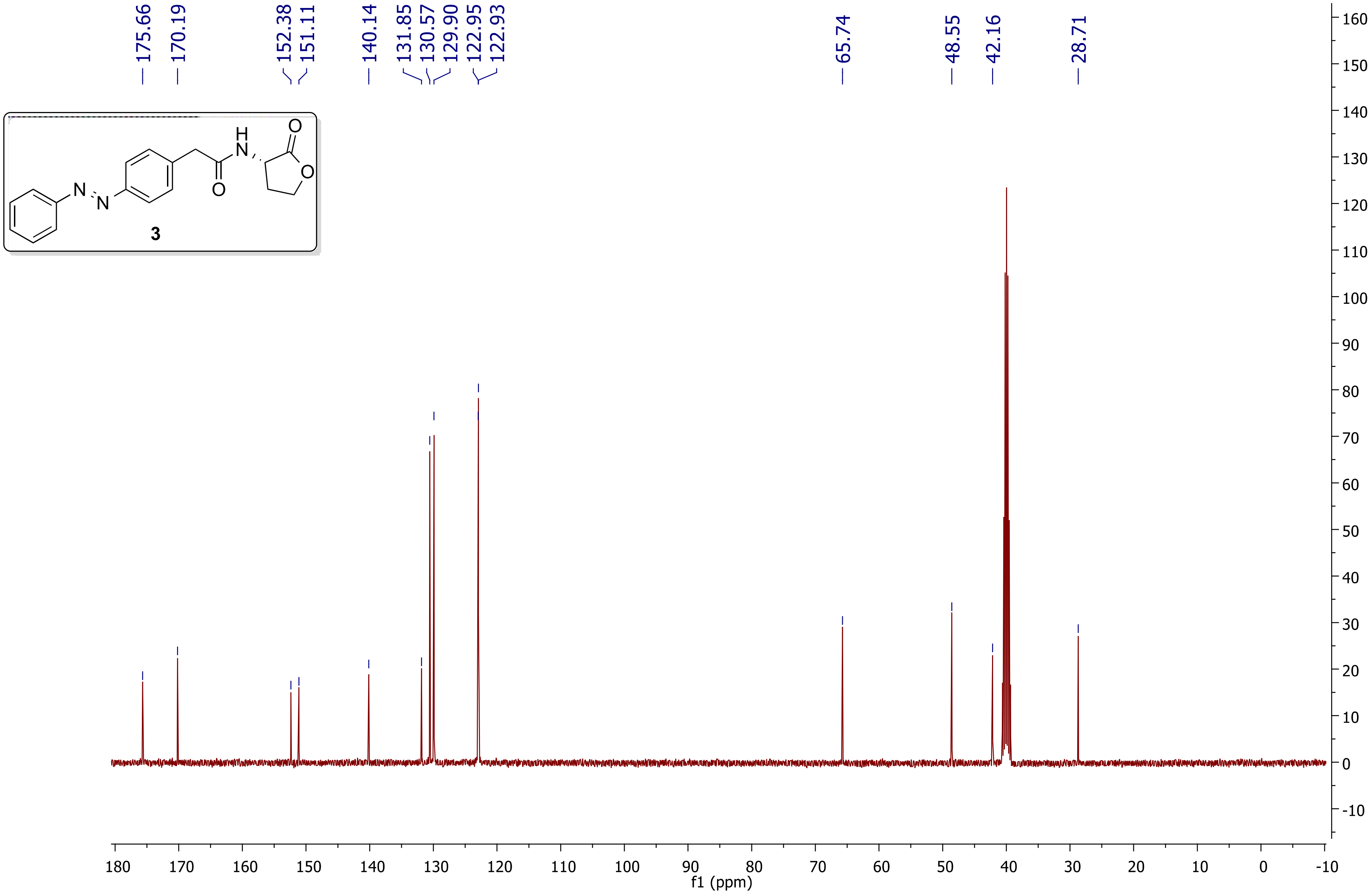
175.66
170.19
152.38
151.11
140.14
131.85
130.57
129.90
122.95
122.93
65.74
48.55
42.16
28.71
3
f1 (ppm)

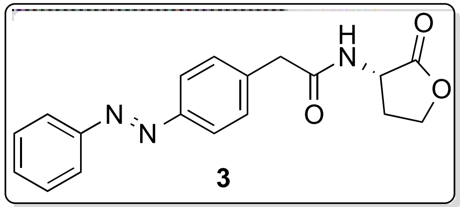

3

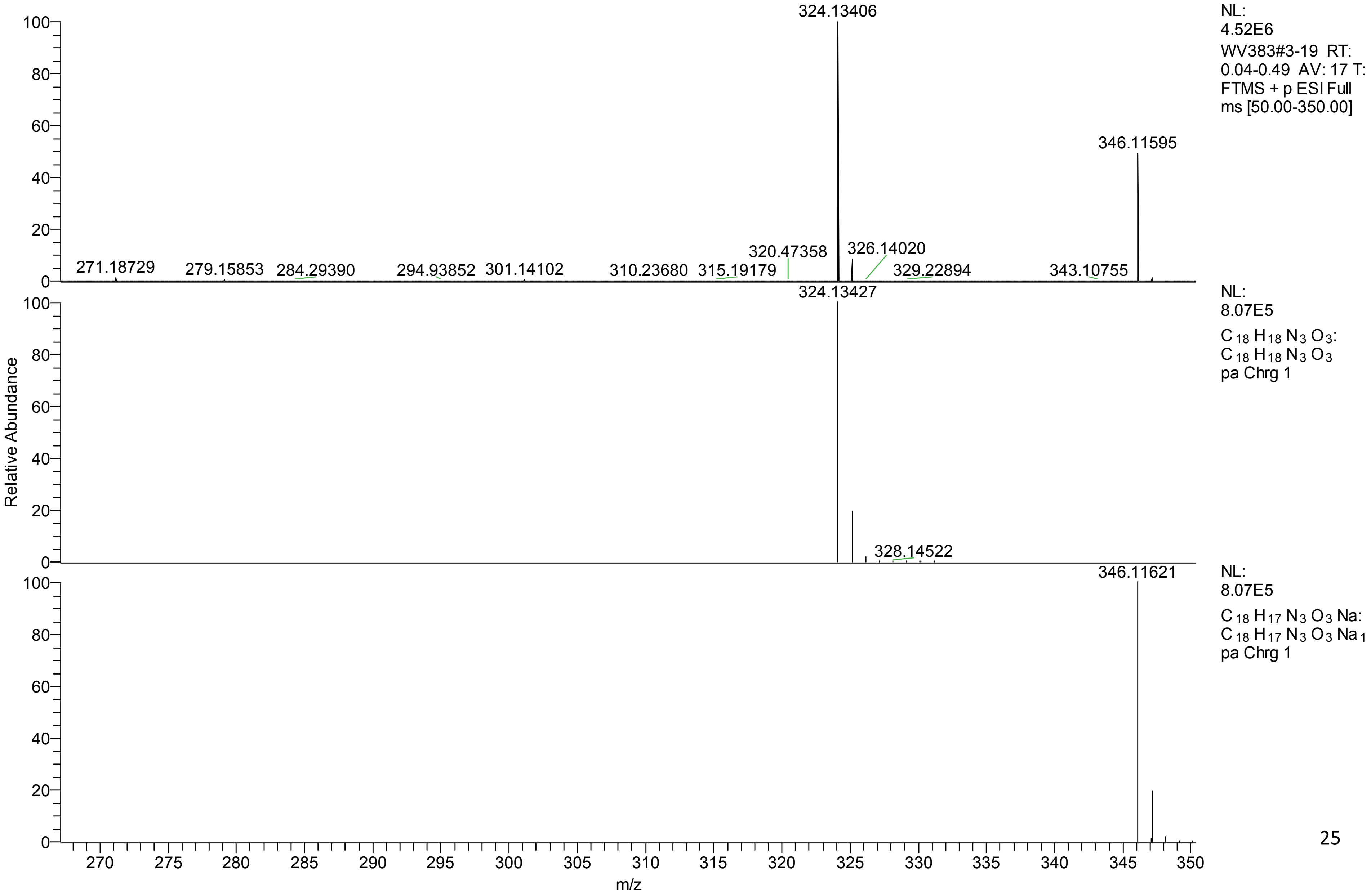
324.13406
NL:
4.52E6
WV383#3-19 RT:
0.04-0.49 AV: 17 T:
FTMS + p ESI Full
ms [50.00-350.00]
346.11595
271.18729
279.15853
284.29390
294.93852
301.14102
310.23680
315.19179
320.47358
326.14020
329.22894
343.10755
324.13427
NL:
8.07E5
C 18 H 18 N 3 O 3:
C 18 H 18 N 3 O 3
pa Chrg 1
328.14522
346.11621
NL:
8.07E5
C 18 H 17 N 3 O 3 Na:
C 18 H 17 N 3 O 3 Na 1
pa Chrg 1
Relative Abundance
m/z
0
20
40
60
80
100
270
275
280
285
290
295
300
305
310
315
320
325
330
335
340
345
350

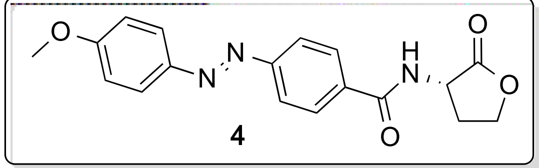
4

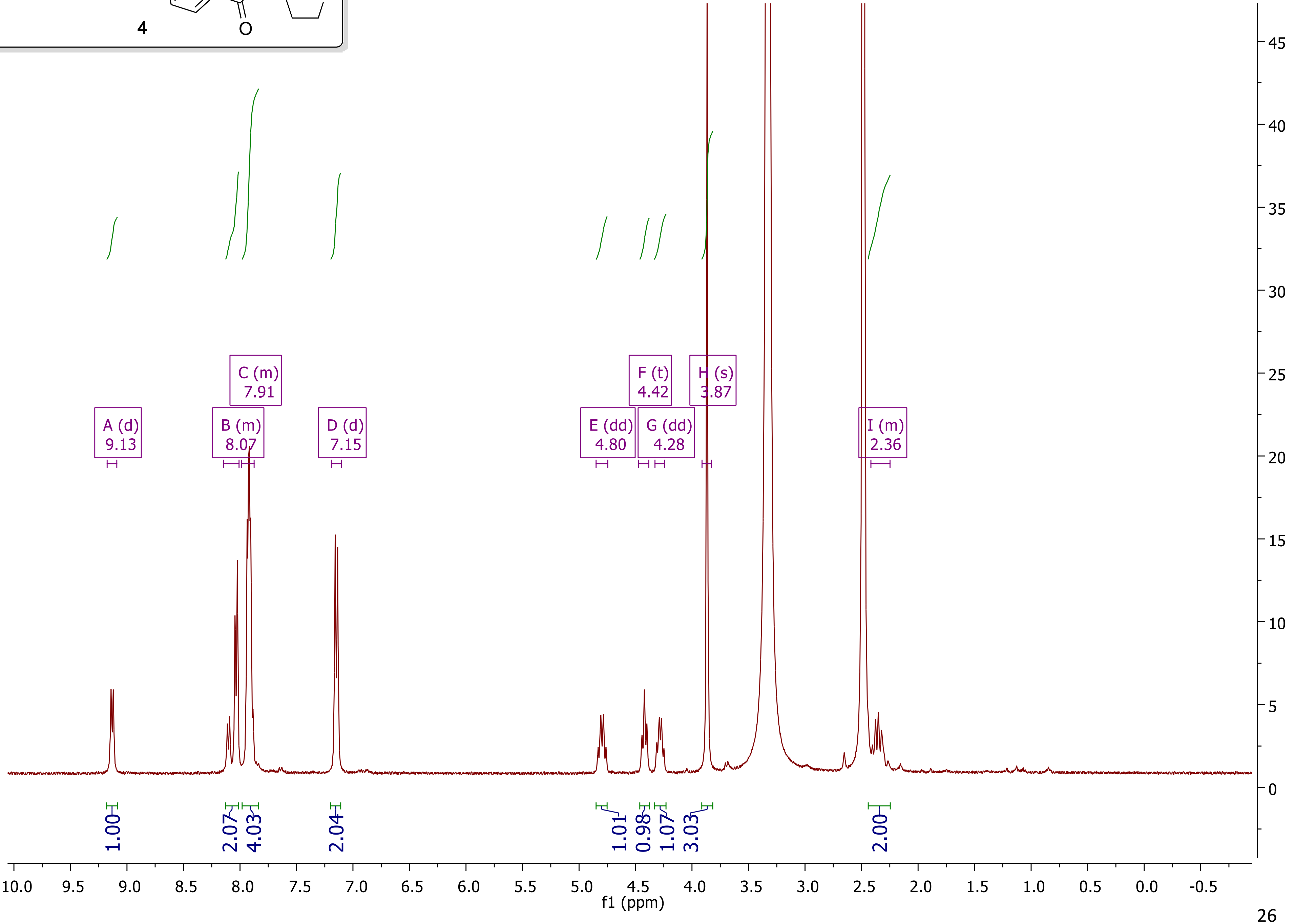
A (d)
9.13
B (m)
8.07
C (m)
7.91
D (d)
7.15
E (dd)
4.80
F (t)
4.42
G (dd)
4.28
H (s)
3.87
I (m)
2.36
1.00
2.07
4.03
2.04
1.01
0.98
1.07
3.03
2.00
f1 (ppm)

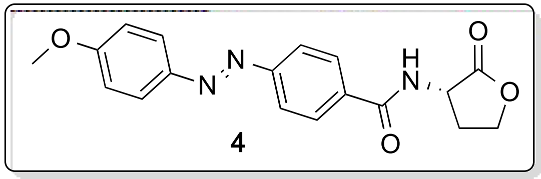
4

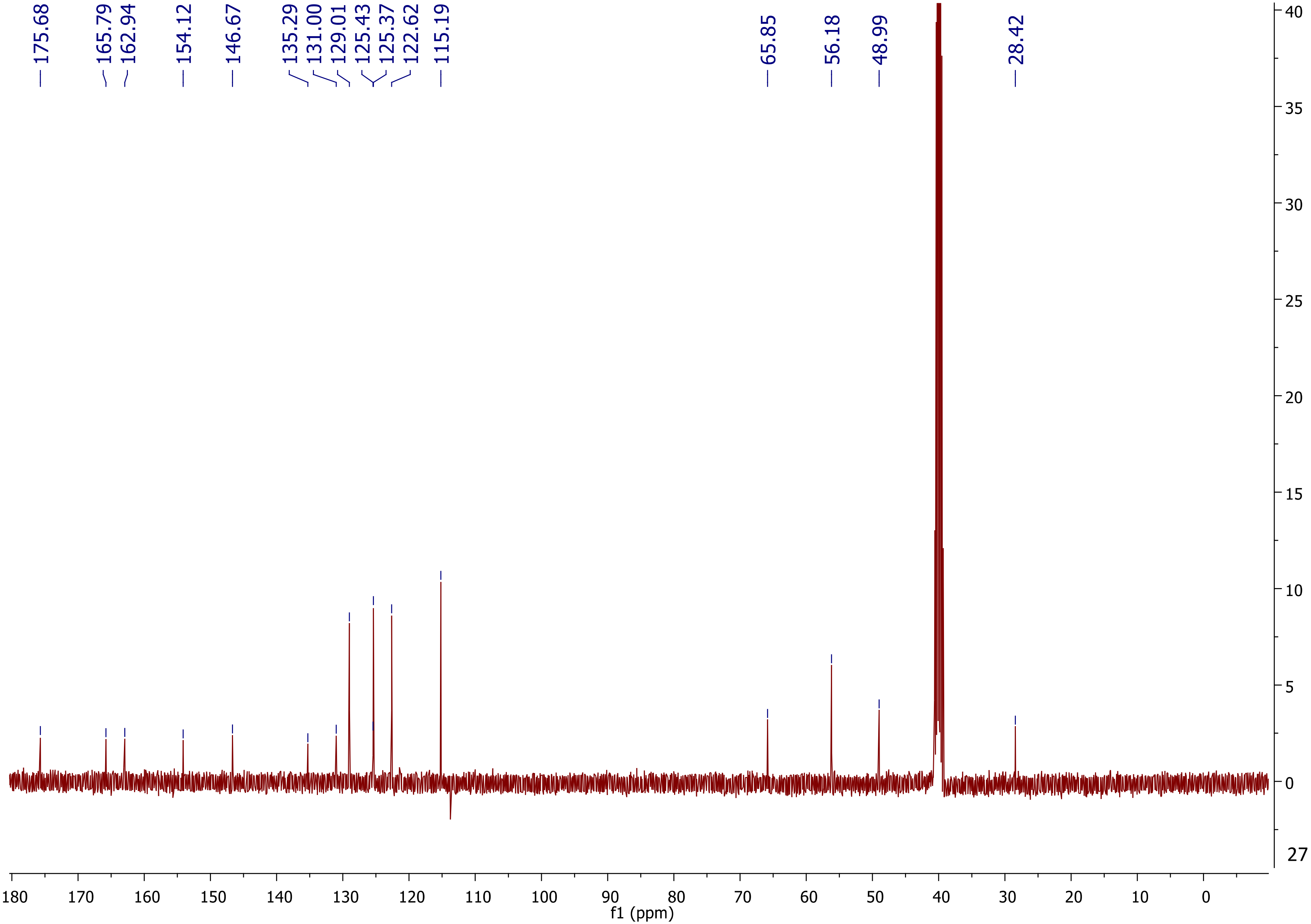
175.68
165.79
162.94
154.12
146.67
135.29
131.00
129.01
125.43
125.37
122.62
115.19
65.85
56.18
48.99
28.42
40
35
30
25
20
15
10
5
0
180 170 160 150 140 130 120 110 100 90 80 70 60 50 40 30 20 10 0
f1 (ppm)

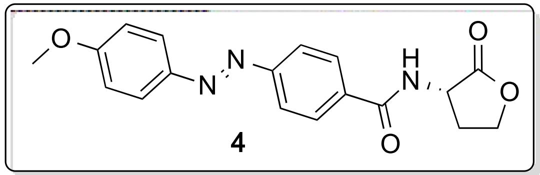

4

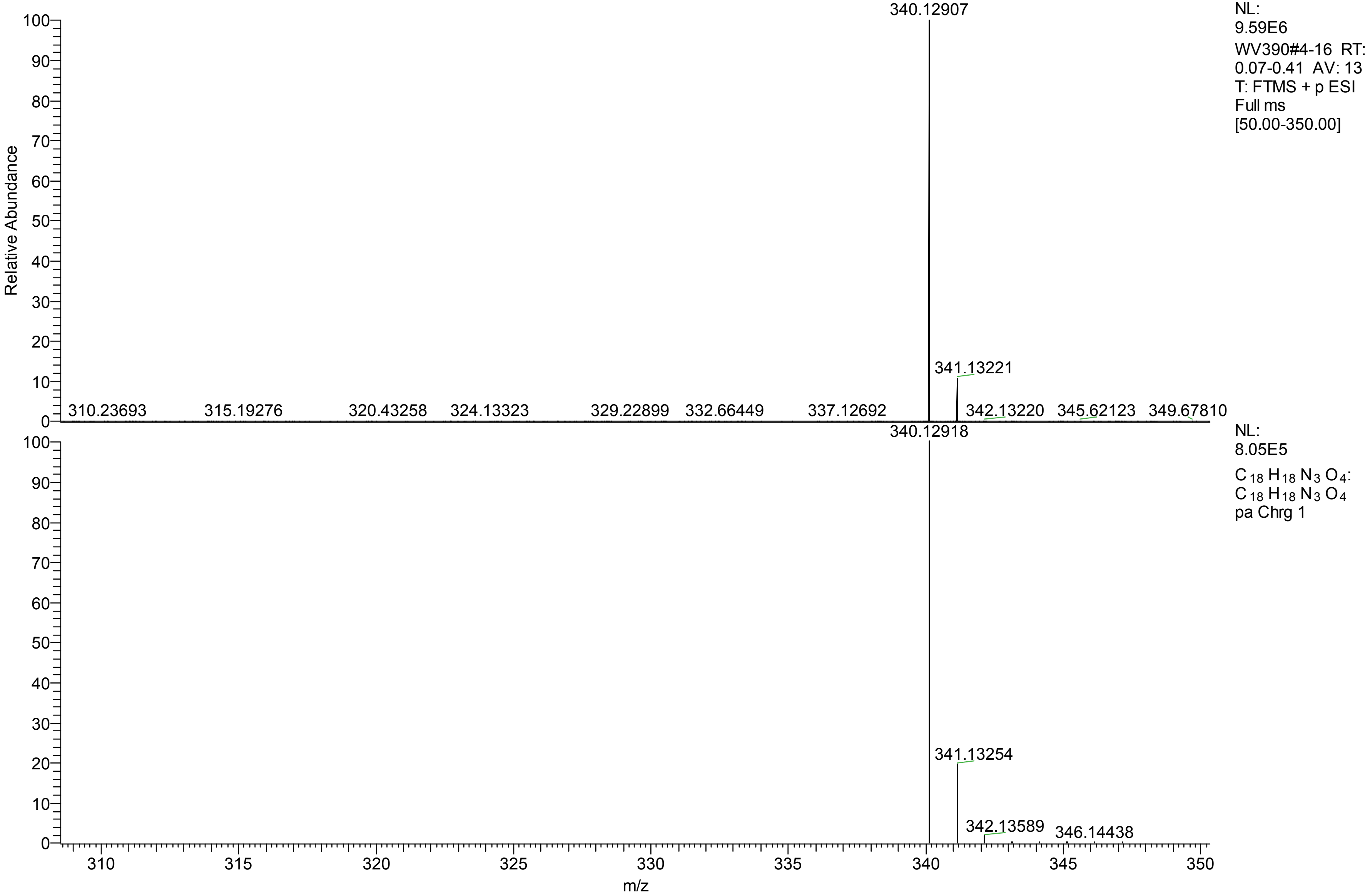

340.12907
NL:
9.59E6
WV390#4-16 RT:
0.07-0.41 AV: 13
T: FTMS + p ESI
Full ms
[50.00-350.00]
Relative Abundance
341.13221
310.23693
315.19276
320.43258
324.13323
329.22899
332.66449
337.12692
342.13220
345.62123
349.67810
340.12918
NL:
8.05E5
C 18 H 18 N 3 O 4:
C 18 H 18 N 3 O 4
pa Chrg 1
341.13254
342.13589
346.14438
m/z

References

1. N. T. Liberati, J. M. Urbach, S. Miyata, D. G. Lee, E. Drenkard, G. Wu, J. Villanueva, T. Wei, and F. M. Ausubel, *Proc. Natl. Acad. Sci. U. S. A.*, 2006, **103**, 2833–2838.

2. M. K. Winson, S. Swift, L. Fish, J. P. Throup, F. Jørgensen, S. R. Chhabra, B. W. Bycroft, P. Williams, and G. S. A. B. Stewart, *FEMS Microbiol. Lett.*, 1998, **163**, 185–192.

3. A. Price-Whelan, L. E. P. Dietrich, and D. K. Newman, *J. Bacteriol.*, 2007, **189**, 6372–6381.

4. J. T. Hodgkinson, W. R. J. D. Galloway, M. Casoli, H. Keane, X. Su, G. P. C. Salmond, M. Welch, and D. R. Spring, *Tetrahedron Lett.*, 2011, **52**, 3291–3294.

5. P. Fatás, E. Longo, F. Rastrelli, M. Crisma, C. Toniolo, A. I. Jiménez, C. Cativiela, and A. Moretto, *Chem. – A Eur. J.*, 2011, **17**, 12606–12611.

6. T. Stafforst and D. Hilvert, *Angew. Chem. Int. Ed. Engl.*, 2010, **49**, 9998–10001.

7. G. A. Molander and L. N. Cavalcanti, *J. Org. Chem.*, 2012, **77**, 4402–13.

8. Y.-K. Lim, K.-S. Lee, and C.-G. Cho, *Org. Lett.*, 2003, **5**, 979–982.

9. K. Kreger, P. Wolfer, H. Audorff, L. Kador, N. Stingelin-Stutzmann, P. Smith, and H.-W. Schmidt, *J. Am. Chem. Soc.*, 2010, **132**, 509–516.
